# The International Match Calendar in Men's Professional Football: An Expert Position Statement

**DOI:** 10.1111/sms.70163

**Published:** 2025-11-06

**Authors:** Steve Den Hollander, Darren Burgess, Gino Kerkhoffs, Vincent Gouttebarge

**Affiliations:** ^1^ Football Players Worldwide (FIFPRO) Hoofddorp the Netherlands; ^2^ Division of Physiological Sciences and Health Through Physical Activity, Department of Human Biology, Faculty of Health Sciences, Lifestyle and Sport Research Centre University of Cape Town Cape Town South Africa; ^3^ Adelaide Football Club Adelaide South Australia Australia; ^4^ Department of Orthopedic Surgery and Sports Medicine Amsterdam UMC location University of Amsterdam Amsterdam the Netherlands; ^5^ Academic Center for Evidence‐Based Sports Medicine (ACES), Amsterdam the Netherlands; ^6^ Aging & Vitality, Musculoskeletal Health, Sports Amsterdam Movement Sciences Amsterdam the Netherlands; ^7^ Amsterdam Collaboration on Health & Safety in Sports (ACHSS), IOC Research Center of Excellence Amsterdam the Netherlands; ^8^ Section Sports Medicine, Faculty of Health University of Pretoria Pretoria South Africa

**Keywords:** Delphi study, fixture congestion, rest, travel, workload

## Abstract

Given the challenges and concerns associated with the congested schedule dictated by the International Match Calendar (IMC), there is a need for a player‐centric expert position statement with specific recommendations and guidance related to the key dimensions of the IMC in men's professional football. Following a review of scientific and occupational health literature and surveys of players and staff in professional football, 19 statements across 6 key dimensions of the IMC were formulated. The statements were then reviewed and refined through a three‐round Delphi study, engaging experts to a consensus‐based position statement. Expert consensus was reached on 12 statements across 5 key dimensions of the IMC: in‐season rest and recovery, off‐season break, mid‐season break, travel, and young player workload. Although consensus was not reached on statements related to match workload, 69% of experts agreed on the need for a maximum limit of matches per player per season. The expert position statement provides recommendations and guidance on structural safeguards within the IMC that prioritize the long‐term health and performance of players.

## 1 Introduction

Professional male footballers face challenges due to the congested schedule dictated by the International Match Calendar (IMC). The number of games players participate in a season varies based on their club's involvements in various competitions and national team responsibilities, often resulting in significant cumulative match exposure for players competing at the highest level [1]. With the reformatting and expansion of major competitions, including the UEFA continental cup competitions, FIFA Club World Cup and FIFA World Cup, these demands continue to grow [1]. This intensified match exposure increases players' susceptibility to injuries, as research shows an incidence rate of 36 injuries per 1000 match playing hours [2].

Studies have also indicated that injury susceptibility increases during periods of fixture congestion, where players have inadequate recovery time between games [3]. Surveys indicate that a significant percentage of professional footballers feel they are playing too many matches with inadequate recovery time, and expressed concerns about the negative impact of long‐haul travel on their recovery, performance and health [4, 5].

Given these challenges and concerns, there is a need for a player‐centric position statement with specific recommendations and guidance related to the key dimensions of the IMC in men's professional football. These recommendations and guidance should prioritize player welfare and injury prevention, with a focus on schedule considerations to allow adequate recovery time between matches, addressing the impact of travel on players, optimizing working conditions, and managing overall workload. This article describes the evidence‐based process followed to define a player‐centric expert position statement related to the key dimensions of the IMC in men's professional football, with the ultimate aim to safeguard the players' physical and mental well‐being.

## 2 Methodological Approach

A modified Delphi survey design was used to develop the position statement, relying on evidence triangulation coming from the following three evidence streams:

1. A scoping review of sports & exercise medicine literature.
2. A systematic literature search and narrative review of occupational and legal safety literature.
3. Survey data collected from professional players and support staff.

### 2.1 Review of the Sports and Exercise Medicine Literature

A scoping review of the scientific literature was conducted (checklist provided in Supporting Information File E), searching in titles and abstracts of the electronic databases MEDLINE (via PubMed) and SPORTDiscus (via EBSCO) from 2000 to August 2024. We applied the following filters (if applicable): Humans; English; Academic journals. A highly sensitive search strategy was built based on 2 groups of keywords: “football” or “soccer”, and “match congestion”, “fixture congestion”, “recovery”, “rest”, “break”, “match workload”, “regeneration”, or “travel” (Supporting Information File A). Within each group of keywords, the search terms were combined by the Boolean command OR, and the 2 groups of keywords were linked by the Boolean command AND. Existing medical subject headings [MeSH] were used if possible, and search terms were truncated with *. Eligibility criteria for inclusion were:

1. The study population consists exclusively of professional male footballers (defined as those playing in international, continental and/or highest or second highest nation competitions).
2. The article presents an original quantitative study.
3. The study outcome is related to one of the following dimensions of the IMC: fixture congestion, midseason break, rest, recovery and/or fatigue, and travel.
4. The article is written in English.

To identify potentially relevant articles, titles and abstracts were screened by 1 researcher (SDH) using the eligibility criteria, the procedure being checked independently by a second researcher (VG). If the title and abstract did not provide sufficient information to determine whether the eligibility criteria were met, they were included for full text review. Then, all full text articles were screened by 1 researcher (SDH) using the eligibility criteria, the procedure being checked independently by a second researcher (VG). To avoid missing any relevant publications, the references of the included studies were screened as well. Any disagreements regarding the inclusion or exclusion of articles were resolved via consensus.

Data from the included articles was extracted by 1 researcher (SDH), the procedure being checked independently by a second researcher (VG). Four standardized extraction forms (one for each key dimension under study) were used to report the following: study information (author, year); study population (e.g., sample size, age, country, level of competition); study design and related information if applicable (e.g., response rate, follow‐up period); outcome (e.g., risk of injury).

### 2.2 Review of the Occupational and Legal Safety Literature

A systematic literature search and narrative review of the scientific literature was conducted to explore the demands, resources, and potential health consequences for professional footballers (Supporting Information File B). An electronic search was conducted in the electronic databases MEDLINE (via PubMed) and Scopus for articles published up to June 2024. Eligibility criteria for inclusion were:

1. Written in English.
2. Categorized as a review, systematic review, or meta‐analysis.
3. Focus on professional football players.
4. Provide data on demands, resources, or potential negative health outcomes.

For studies involving a mixed population of professional, semi‐professional, and/or amateur players, only data from the professional players was extracted. Data from the included studies were extracted systematically, and categorized into general study details, research objectives, key findings, and their alignment with the Job Demands–Resources model [6].

### 2.3 View of Players and Staff Members

Two cross‐sectional observational studies by means of an electronic survey were conducted among elite male professional footballers in order to explore their views and perceptions on various key dimensions of the IMC [4, 5]. Recruited by FIFPRO (Football Players Worldwide) and its national members, players met the following inclusion criteria: (i) male; (ii) age 18 or older; (iii) professional footballer playing or had played in international, continental and/or national (highest or second highest) competition with his club and/or national team; (iv) able to read and comprehend texts fluently in English, French, or Spanish. Two electronic surveys (available in English, French and Spanish) were designed during expert meetings with football stakeholders (governing bodies, players' unions, current/retired players), and included questions related to various key dimensions of the IMC (e.g., influence of traveling, in‐ and off‐season breaks). Players were asked to give their informed consent (electronically) and to anonymously complete their survey (FluidSurveysTM, Ottawa, Canada) within 2 weeks. Once completed (around 10 min was needed), electronic surveys were saved automatically on a secured electronic server.

A third and last cross‐sectional observational study by means of an electronic survey was conducted among staff members in order to explore their views and perceptions on various key dimensions of the IMC [7]. Staff members met the following inclusion criteria: (i) age 18 or older; (ii) working as staff members in the medical, performance and/or strength and conditioning team in professional football (club and/or national team); (iii) able to read and comprehend texts fluently in English. An electronic survey (available in English) was designed, including questions related to various key dimensions of the IMC (e.g., number of matches and related recovery, season preparation). Staff members were asked to give their informed consent (electronically) and to anonymously complete their survey (FluidSurveysTM, Ottawa, Canada) within 2 weeks. Once completed (around 10 min was needed), electronic surveys were saved automatically on a secured electronic server.

### 2.4 Consensus Towards Position Statement

#### 2.4.1 Study Design

This study used a modified three‐round Delphi survey design, conducted in accordance with the Conducting and Reporting Delphi Studies (CREDES) guidelines [8]. The modified Delphi technique enables expert knowledge collection through multiple rounds of questionnaires, that include written responses, to reach consensus about a specific topic. This method relies on anonymity, iteration, controlled feedback, and statistical aggregation of responses [9]. The modified Delphi technique is widely recognized and has become increasingly popular in sports and exercise medicine [10, 11, 12]. The survey was conducted in English. Ethical approval for the study was obtained from the Medical Ethics Review Committee of the Amsterdam University Medical Centers (2024‐0970), and the study was conducted in accordance with the principles set out in the Declaration of Helsinki (2024).

#### 2.4.2 Experts

Experts meeting the following inclusion criteria were invited to participate: (i) working in performance or medical roles within men's professional football (at club or national team level) and (ii) a minimum age of 18 years. Members of FIFPRO's High‐Performance Advisory Network (*n* = 12) were asked to recommend experts who met the inclusion criteria. Experts were recruited using convenience sampling, with the participation of at least 50 experts as a target [13].

#### 2.4.3 Key Dimensions of the IMC

Informed by the scientific literature review (2.1 step 1 and 2.2 step 2) and the players' and staff members' views (2.3 step 3), and in consultation with FIFPRO's High‐Performance Advisory Network, 6 key dimensions of the IMC were identified and defined:

1. Match workload
  - Total number of matches: the number of times a player played at least 45 min in a competitive match (club and national team) over a single season.
  - Total number of appearances: the number of times a player played any minutes in a competitive match (club and national team) over a single season.
  - Total number of squad inclusions: the number of times a player was part of a competitive matchday squad, whether they played any minutes or were an unused substitute (club and national team) over a single season.
  - Back‐to‐back appearances: If a player did not have at least 5 days of rest and recovery time between two appearances.
2. In Season Recovery and Fatigue
  - Rest between matches: the rest period (in calendar days) between 2 matches.
3. Off‐season break
  - Off‐season break: the number of rest days without any football activity in the club environment between 2 seasons to recover and regenerate.
  - Blackout period: A period within the off‐season break with no communication from club or national team.
4. In‐season break
  - In‐season break: the number of rest days without any football activity in the club environment during a season to recover and regenerate.
5. Travel
  - Long‐haul flight: flight that exceeds 6 h.
6. Young player workload safeguards

#### 2.4.4 Procedure

Based on the 6 key dimensions of the IMC, 19 statements were developed for the position statement (Figure 2) by the authors in consultation with FIFPRO's High‐Performance Advisory Network. Potential participants received information about the study via email. If interested in the study, all participants gave their informed consent and received access to the first of the 3 surveys (all set up in English within CastorEDC, CIWIT B.V, Amsterdam, The Netherlands). Each survey round was open for 2 weeks, with a single reminder sent after 1 week. The three‐round Delphi study took place from February to March 2025.

#### 2.4.5 Round 1

The first round of surveys included 19 statements. Participants rated their level of agreement on a 5‐point Likert scale (strongly agree, agree, neutral, disagree, strongly disagree). A consensus threshold was set at 75% agreement (i.e., “agree” or “strongly agree”) or disagreement (i.e., “disagree” or “strongly disagree”) [14]. For statements where participants disagreed or strongly disagreed, follow‐up questions were provided to seek clarity on whether:

- They agreed with the statement but believed the number in the statement should be different (if applicable).
- They agreed with the statement but felt there was insufficient research to support it.
- They disagreed with the statement.

Open text boxes were provided to allow participants to provide additional comments. Statements that reached consensus were included in the final position statement and not included in the subsequent rounds. Statements with a majority (> 50%) agreement or disagreement advanced to Round 2. Statements with less than 50% majority agreement (agree or disagree) were removed from the position statement. The feedback from the follow‐up questions and open text boxes was reviewed to determine if any additional statements needed to be added to Round 2.

#### 2.4.6 Round 2

In Round 2, participants indicated their level of agreement using a 4‐point Likert scale (strongly agree, agree, disagree, strongly disagree). Respondents who disagreed (or strongly disagreed) with a statement were asked the same follow‐up questions as in Round 1 for clarification. Open‐text boxes were again provided for additional comments.

Statements that reached consensus were included in the final position statement. Statements where at least two‐thirds (> 66%) of experts agreed or disagreed were rephrased for Round 3, based on the responses to the follow‐up questions and qualitative data. Statements with less than a two‐thirds majority agreement (agree or disagree) were removed from the position statement.

#### 2.4.7 Round 3

As in Round 2, respondents indicated their level of agreement using a 4‐point Likert scale (strongly agree, agree, disagree, strongly disagree). No follow‐up questions or open text boxes were provided. Statements that reached consensus were included in the final position statement, while those failing to reach consensus were removed.

#### 2.4.8 Data Analysis

After each round, results were analyzed prior to the development and distribution of subsequent rounds. Descriptive analyses (e.g., mean, percentages) assessed whether statements reached consensus. Qualitative analysis of open‐text responses was performed after each round by 2 researchers (SDH, VG) to identify themes and expert suggestions. Descriptive analyses were performed to summarize the participants' demographics and professional experience.

## 3 Results

### 3.1 Review of the Sports and Exercise Medicine Literature

**FIGURE 1:**
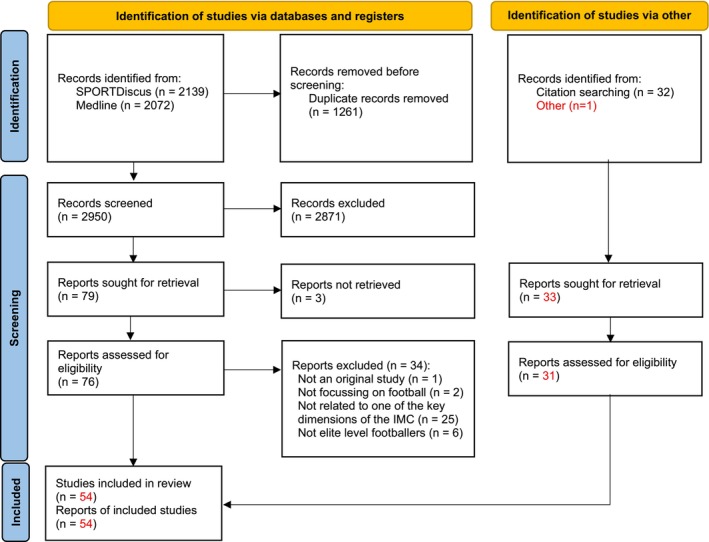
PRISMA flow diagram of literature search.

A total of 4211 potentially relevant citations were retrieved. After removing 1261 duplicates, 2950 citations remained. After applying the inclusion criteria to the titles and abstracts, 79 potentially relevant studies were included for full text review. From these, 37 studies were excluded for various reasons (not an original study: *n* = 1; not focusing on football: *n* = 2; not related to one of the key dimensions of the IMC: *n* = 25; sample was not elite professional footballers: *n* = 6). The reference check of the included studies resulted in 12 additional relevant studies. Consequently, 54 original studies were finally included. The PRISMA flow chart of the search procedure is presented as Figure 1. From the included studies, 4 key dimensions of the IMC were identified: fixture congestion, rest, recovery and fatigue, midseason break, and travel.

Seventeen studies reported the effect of fixture congestion on injuries, with 10 reporting an association between an overload of match workload and injuries (59%) [15, 16, 17, 18, 19, 20, 21, 22, 23, 24] and 1 reporting an association between an underload of match workload and injuries (6%) [25]. All nine studies examining the effects of fixture congestion on health and wellness outcomes reported significant associations. These outcomes included fatigue [26, 27, 28, 29, 30], muscle soreness [26, 28, 31], biochemical stress markers [29, 31, 32], immune suppression [33], inflammation [34], and mood disturbances [32]. One out of 10 studies (10%) [35] found that fixture congestion negatively impacted performance (Table SC1) [15, 16, 17, 18, 19, 20, 21, 22, 23, 24, 25, 26, 27, 28, 29, 30, 31, 32, 34, 35, 36, 37, 38, 39, 40, 41, 42, 43, 44, 45].

There were 7 studies related to rest, recovery and/or fatigue. Three studies (43%) [46, 47, 48] showed that at least 72 h are required to fully recover physically from a match (Table SC2) [22, 30, 33, 46, 47, 48, 49].

Five studies described the effect of a midseason break on injuries or performance (Table SC3). Two out of 3 studies found that a break would reduce the risk of injury [50, 51], and 100% of studies found that a break would have a positive impact on performance [44, 50, 51, 52, 53].

Nine studies assessed the impact of travel on either health/wellness, injuries and/or performance (Table SC4). One study found that international travel increased the risk of injury (100%) [17], with 4 studies reporting a negative impact on health/wellness (100%) [54, 55, 56, 57]. Additionally, 4 out of 5 studies (80%) [54, 58, 59, 60] found that international travel had a negative impact on match performance [17, 54, 55, 57, 58, 59, 60, 61, 62].

Seven cross‐sectional surveys were identified in this review, with players (*n* = 4) and practitioners (*n* = 3) surveyed regarding key indicators of the IMC including fixture congestion (*n* = 4), rest and recovery (*n* = 3), fatigue (*n* = 3), and international travel (*n* = 2). In all 7 survey studies, the indicators of the IMC surveyed were identified by the players and medical practitioners as key risk factors for injuries (Table SC5) [4, 5, 63, 64, 65, 66, 67].

### 3.2 Review of the Occupational and Legal Safety Literature

Thirty‐seven articles were included in the review. From an occupational perspective, the literature showed that high match frequency, inadequate recovery time, and intense physical demands pose serious risks to the players' health. Findings showed that the majority of the footballers reported injuries from overloaded match schedules. Around 40% experienced negative mental health effects, exacerbated by travel, frequent competition, and limited off‐season breaks. Mental and physical fatigue often lead to burnout, anxiety, and depression, all of which should be recognized as occupational injuries. Additionally, new competition formats (e.g., FIFA World Cup expansion) and congested calendars increase workload, further intensifying these risks. This calls for enhanced protective regulations in line with Occupational Safety and Health (OSH) principles that prioritize long‐term health over short‐term performance. A comprehensive risk management strategy is recommended, requiring clubs to assess job content, work relations, work environment, and organizational aspects to mitigate risks and support player health. Integrating OSH practices within football institutions benefits player welfare, reduces injury risks, and fosters sustainable careers.

From a legal standpoint, footballers are recognized as employees and must be protected under labour laws. International frameworks, such as the International Labour Organization (ILO) conventions, European Social Charter (ESC), and EU regulations, require that clubs and governing bodies implement OSH standards to safeguard the players' well‐being. This includes not only physical demands but also the mental health demands, employment terms, and work relations. The full report on the review of occupational and legal safety literature in football can be found in Supporting Information File B.

### 3.3 View of Players and Staff Members

A total of 543 players completed the first survey and 1055 players the second. One‐third of the players reported playing too many matches per season, having negative consequences on their performance and/or health. Nearly 75% of the players felt that 3 to 4 back‐to‐back matches were the maximum number of matches that should be allowed, while 40% reported that the number of recovery days between matches was insufficient to recover, perform optimally and/or to avoid injuries. Around 35% of the players indicated they had sustained an injury due to match congestion. Nearly all players (85%) were in favor of an in‐season break lasting 14 days (SD = 6) in order to recover from the first half of the season. Players reported that the off‐season break should last 5 weeks (SD = 3) in order to recover and regenerate physically and mentally before the start of a new season. Nearly two‐thirds mentioned that long national and international flights (> 2 h) had a significant negative impact on their recovery, performances and/or health. Detailed information on the players' views and perceptions is described elsewhere [4, 5, 7].

A total of 92 staff members completed the survey (most based in Europe), 27% being high performance coaches, 21% sports scientists, 16% strength and conditioning coaches, 15% physical therapists, and 9% medical doctors. Nearly 70% of the staff members felt that 40 to 54 matches per season should be played, while 60% felt that 3 to 4 back‐to‐back matches were the maximum number of matches that should be allowed. Nearly all staff members (85%) were in favor of an off‐season break lasting 4 to 6 weeks, while the large majority indicated that 6 weeks was needed to prepare players for the start of the football season [7].

### 3.4 Consensus Towards Position Statement

#### 3.4.1 Participants

A total of 133 experts were invited to participate in the study, with 70 agreeing to take part (53% response rate). Of these, 55 completed Round 1 (79% completion rate), 55 completed Round 2 (79% completion rate), and 63 completed Round 3 (90% completion rate). The collective experience of the expert group is provided in Table 1.

**TABLE 1:** Experts' characteristics.

Characteristics	%
*Age*	
18–30	2
31–40	27
41–50	42
51–60	17
> 60	12
*Role*	
High Performance	36
Medical	64
*Primary involvement*	
Club	73
National Team	27
*Confederation*	
AFC	7
CAF	7
CONCACAF	12
CONMEBOL	0
OFC	0
UEFA	74
*Experience*	
0–10 years	22
11–20 years	54
21–30 years	17
> 30 years	7

#### 3.4.2 Round 1

**FIGURE 2:**
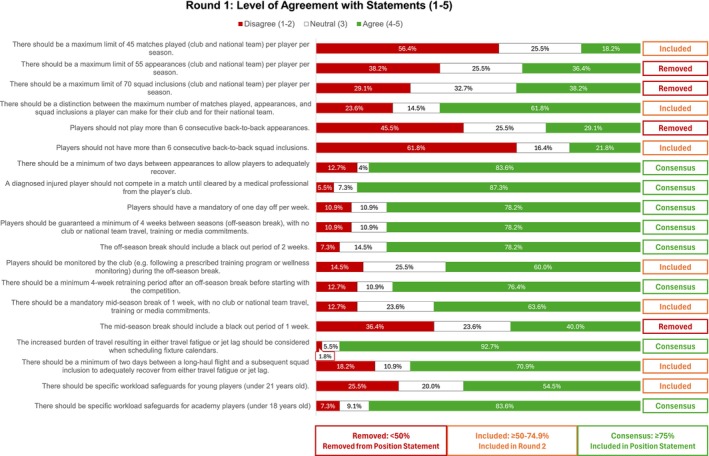
Statements and agreement ratings for Round 1.

The results of Round 1 are presented in Figure 2. Eight statements reached consensus and were included in the final position statement. Seven statements, with over 50% agreement or disagreement, advanced to Round 2. Four statements, receiving less than 50% agreement in any category, were removed from the position statement.

On the key dimension of back‐to‐back appearances, experts suggested focusing on fixture density rather than a strict four‐day interval between matches. As such, a new statement was developed and included in Round 2: “Players should not make more than 2 appearances per week for more than 3 consecutive weeks (6 appearances over 3 weeks).”

#### 3.4.3 Round 2

**FIGURE 3:**
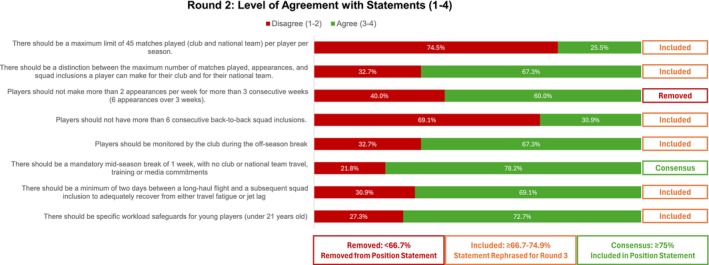
Statements and agreement ratings for Round 2.

Figure 3 presents the results of Round 2. One statement reached consensus and was included in the final position statement. One statement, with less than 67% agreement in any category, was removed from the position statement. The remaining 6 statements, with over 66% agreement or disagreement, were rephrased based on the follow‐up questions and qualitative feedback in Round 2, before progressing to Round 3 (Supporting Information File D).

#### 3.4.4 Round 3

**FIGURE 4:**
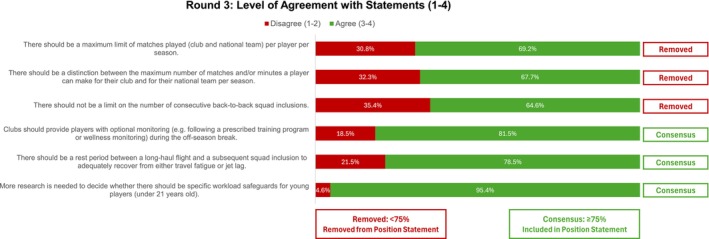
Statements and agreement ratings for Round 3.

Three statements reached consensus and were included in the final position statement, while 3 did not and were removed (Figure 4).

#### 3.4.5 Final Position Statement

Elaborating on 5 key dimensions of the IMC, the expert position statement in men's professional football finally comprised 12 statements, namely:

- *In Season Rest & Recovery*: There should be a minimum of 2 days between appearances to allow players to adequately recover.
- *In Season Rest & Recovery*: A diagnosed injured player should not compete in a match until cleared by a medical professional from the player's club.
- *In Season Rest & Recovery*: Players should have a mandatory of 1 day off per week.
- *Off‐Season Break*: Players should be guaranteed a minimum of 4 weeks between seasons (off‐season break), with no club or national team travel, training or media commitments.
- *Off‐Season Break*: The off‐season break should include a black out period of 2 weeks.
- *Off‐Season Break*: There should be a minimum 4‐week retraining period after an off‐season break before starting with the competition.
- *Off‐Season Break*: Clubs should provide players with optional monitoring (e.g., following a prescribed training program or wellness monitoring) during the off‐season break.
- *Mid‐Season Break*: There should be a mandatory mid‐season break of 1 week, with no club or national team travel, training or media commitments.
- *Travel*: The increased burden of travel resulting in either travel fatigue or jet lag should be considered when scheduling fixture calendars.
- *Travel*: There should be a rest period between a long‐haul flight and a subsequent squad inclusion to adequately recover from either travel fatigue or jet lag.
- *Young Player Safeguards*: There should be specific workload safeguards for academy players (under 18 years old).
- *Young Player Safeguards*: More research is needed to decide whether there should be specific workload safeguards for young players (under 21 years old).

## 4 Discussion

This paper presents the development of an expert position statement on the IMC in professional men's football. The process involved a review of the scientific and occupational health literature and surveys of players and staff in professional football, leading to the formulation of 19 key statements across 6 key dimensions of the IMC. These statements were then reviewed and refined through a three‐round Delphi study, engaging experts to reach a consensus‐based position statement on the IMC in professional men's football. Ultimately, consensus was reached on 12 statements spanning 5 key dimensions: *in‐season recovery and fatigue, off‐season break, mid‐season break, travel*, and *workload safeguards for young players*.

### 4.1 In Season Recovery and Fatigue

Experts reached a consensus that there should be a minimum of 2 days between appearances and that players should have a mandatory day off each week. Additionally, they agreed that a diagnosed injured player should not compete in a match until cleared by a club medical professional.

Research supports these recommendations, with evidence indicating that full recovery from a football match can take between 48 and 72 h (Table SC2). Research has also shown that players in teams with congested match schedules tend to return to play earlier than expected, increasing their risk of subsequent injury [24]. This suggests that match workload, combined with performance pressures, may contribute to premature returns from injury, increasing the risk of subsequent reinjury [24]. Given these findings, it is crucial that return‐to‐play decisions remain the responsibility of medical professionals, in alignment with expert consensus, to safeguard players' long‐term well‐being while balancing competitive demands.

As contracted athletes, professional footballers are employees of their clubs and/or national teams, making them subject to international labour laws and regulations. These regulations include the right to a weekly day off work, free from any work‐related obligations such as travel and media commitments [68]. In alignment with expert consensus, ensuring the consistent implementation and enforcement of this right should be a priority for unions and other key stakeholders in professional football.

### 4.2 Off‐Season Break

Over 75% of experts agreed that there should be a 4‐week off‐season period, free from club or national team travel, training, or media commitments. This period should include a 2‐week black out period with no contact from the club and optional monitoring, followed by a structured 4‐week retraining phase before the start of a new season. However, under the current 2024/25 IMC, implementing this framework appears challenging.

For example, the 2024/25 Premier League and La Liga seasons concluded on 25 May, with the UEFA Champions League Final a week later on 31 May. An international match window was available for all confederations between 2 and 10 June, which included the Nations League finals. The Concacaf Gold Cup was scheduled between 14 June and 6 July, and the FIFA Club World Cup between 15 June and 13 July. With the 2025/2026 Premier League and La Liga seasons set to start on 16 August—less than 5 weeks after the conclusion of the FIFA Club World Cup—players will either have less than 4 weeks to recover from the previous season (off‐season), or less than 4 weeks to prepare for the next season (preseason retraining phase).

To safeguard players, measures need to be put in place to allow sufficient time for recovery and preparation between seasons, balancing commercial and competitive demands with the long‐term health and performance of professional footballers.

### 4.3 Mid‐Season Break

Experts reached consensus on the need for a mandatory one‐week mid‐season break, with no club or national team travel, training, or media commitments. This recommendation aligns with research showing that mid‐season breaks reduce injury risk [51]. Research also indicates that this break should not exceed one week, as longer mid‐season breaks have been associated with reduced physical and technical match performance [52, 53].

To align with the expert consensus on both mid‐ and off‐season breaks, the IMC should be structured over a 44‐week schedule, incorporating a one‐week rest period midway. This would allow players adequate time for physical and mental recovery throughout and between seasons, promoting long‐term well‐being and sustained performance.

### 4.4 Travel

Ninety‐two percent of experts agreed that the burden of travel should be considered when scheduling fixture calendars, and 78% agreed that there should be a rest period between a long‐haul flight and subsequent squad inclusion. Research indicated that frequent travel negatively impacts health, wellness, and performance, while also increasing injury risk (Table SC4). From an occupational health perspective, frequent long‐distance travel has been identified as a potential risk factor for mental health concerns. Surveyed players also reported that flights exceeding 2 h negatively impacted their recovery, performance and overall health.

To mitigate these challenges, safeguards should be integrated into fixture calendar scheduling to reduce the cumulative burden of travel. Strategies could include fewer but longer international fixture windows to reduce the total travel time, as well as policies preventing the scheduling or rescheduling of fixtures within 3 days of international or certain continental matches. Implementing such measures would help balance competition demands with player welfare, ensuring that both physical and mental recovery remain a priority in an increasingly congested football calendar.

### 4.5 Young Player Workload Safeguards

The majority of experts supported the need for specific workload safeguards for young players, with 84% in favour of specific workload safeguards for players under 18 and 73% for players under 21. This reflects growing concern over the increasing demands placed on young footballers at an early stage in their careers [1, 69].

A recent report highlighted that leading under 21 players from Germany, Spain and England are playing considerably more minutes than their predecessors did at the same age. This trend was highlighted as a potential risk to both immediate health and long‐term career longevity of young players [1].

While consensus was reached on the need for specific safeguards for under 18 players, 95% of experts agreed that more research was needed to decide whether there should be specific workload safeguards for under 21 players. Only 6% of studies identified in the sports and exercise medicine literature review included samples with an average age of under 21, indicating a notable gap in the evidence base. As young players are increasingly exposed to senior professional men's football [1], it is important that tailored, evidence‐based safeguards are developed to support their long‐term development, health, and performance.

### 4.6 Match Workload

Although none of the statements related to Match Workload reached consensus, 69% of experts agreed on the need for a maximum limit of matches per player per season (Figure 4). However, the question of how many matches that limit should be remains a complex discussion among experts. Only 25.5% supported a limit of 45 matches played (defined as > 45 min played per match) (Figure 3), while 36.4% agreed that the maximum number of appearances (any minutes played) should be 55 (Figure 2). These perspectives differ from those of surveyed coaches, 87% of whom suggested that the maximum number of matches per season should be below 55. Additionally, 66% of players reported that playing too many matches per season negatively affected their performance and well‐being.

The occupational health and safety review identified high match frequency as an occupational health risk for injuries, reinforcing the occupational health and safety principle to prioritize long‐term health over short‐term performance. However, determining a precise match limit per season remains challenging due to the limited scientific research. The scoping review of the literature found no studies that directly linked the number of matches played in a season to injury risk. One study comparing World Cup players with non‐World Cup players found that, while World Cup participants played significantly more matches throughout the season (46 vs. 33), they did not experience an increased injury risk during the season itself. However, 29% of World Cup players sustained injuries during the tournament, and 32% underperformed, suggesting that the impact of excessive match workload may emerge in subsequent seasons or off‐season tournaments [35]. Further research is needed to explore these potential long‐term impacts.

A number of studies identified acute fixture congestion as a contributing factor for injuries and health and wellness in professional men's football (Table SC1) [3]. However, expert consensus on acute workload thresholds remains mixed. While only 29% of experts agreed that players should not play more than 6 consecutive back‐to‐back matches (Figure 2), 60% agreed that players should not make more than 6 appearances over 3 weeks (Figure 3). In contrast, 75% of players indicated that 3 to 4 back‐to‐back matches should be the maximum allowed. This variation in perspectives between experts and players, alongside scientific evidence, highlights a misalignment in perceived acceptable workload and recovery needs. Such misalignment can compromise player welfare if decisions are based solely on performance goals or subjective perceptions. Therefore, integrating occupational health and safety principles into the development of the IMC can help ensure that player workload is managed within a structured, evidence‐based framework that supports both performance and long‐term well‐being.

### 4.7 Strengths and Limitations

This study used a systematic and transparent approach to develop an expert consensus statement on the IMC in professional men's football. The process was informed by a review of the sports and exercise medicine and occupational health literature and strengthened by the inclusion of the perspectives from both players and staff. The use of a three‐round Delphi study following evidence triangulation added methodological rigour, through an iterative refinement process to progressively build consensus. Inter‐rater dynamics were managed through both quantitative and qualitative processes which limited subjective bias and enabled structured expert feedback. The appropriate sample size [13] and relatively high response attrition rates [70], further strengthen the reliability of the findings.

However, there were several limitations to consider. The views of staff (medical and high‐performance) informed the development of the initial statements for the Delphi Study. Subsequently, medical and high‐performance staff were recruited for the Delphi Study. This may raise concerns about confirmation bias; however, the participants for the survey and the Delphi study were recruited independently from each other, with 3 years between the studies. Both studies were also conducted anonymously, so the authors did not know the views of the individual experts. Furthermore, none of the experts who completed the survey were directly involved in drafting the statements for the Delphi study.

Additionally, the recruitment methods led to some selection bias. The majority of experts (74%) had spent most of their careers in UEFA football, which may limit the generalizability of their views to other regions. As a result, the perspectives captured in the statements may not fully reflect the diversity of professional men's football globally.

## 5 Perspectives

This paper presents a consensus‐driven expert position statement on the IMC in professional men's football, informed by scientific evidence, occupational health principles, and the perspectives of players and coaches. Expert consensus was reached on 12 statements across 5 key dimensions of the IMC: in‐season rest and recovery, off‐season break, mid‐season break, travel, and young player‐specific safeguards. These findings underscore the need for structural safeguards that prioritize long‐term player health and performance, particularly in the face of an increasingly congested match calendar.

While consensus on match workload thresholds was not reached, there was broad agreement on the need to set maximum limits, reflecting growing concern over cumulative workload, injury risk, and the long‐term impact on players' physical and mental health, and performance. Experts reported that insufficient research and too many unknowns made it premature to set specific thresholds. More research, including systematic reviews of existing studies, is needed to assess the quality of available evidence and better inform guidelines for experts working in the field.

A clear call for research targeted at developing specific, evidence‐based workload guidelines for under 21 and under 18 footballers was identified. Young players face distinct physiological, psychological, and social demands compared to established professionals, and poorly managed workloads during these critical stages of growth may have long‐term consequences on their health, performance, and career longevity [71]. Establishing tailored recommendations for rest, recovery, training load, and competition exposure is, therefore, essential to safeguard their development and ensure a safe progression into elite football.

As most of the experts who participated in the study spent the majority of their careers within UEFA, it should be acknowledged that perspectives from other regions may vary. Gathering insights from staff across different confederations could help to better understand potential regional differences. In addition, the player and staff surveys cited in this study [4, 5, 7] were conducted over three years ago. Given that the IMC is a rapidly evolving issue, it is possible that these views have shifted, and updated surveys could be valuable to ensure that current perspectives are accurately reflected.

Meaningful changes to the IMC are needed to promote sustainable careers and optimal performance. This includes adopting evidence‐informed policies related to travel, protecting recovery time, and safeguarding the development of young players. The position statement outlined in this paper offers a foundation for more player‐centered scheduling, balancing the demands of the global game with the fundamental rights and health of those who make the game possible.

## Author Contributions

S.D.H. participated in the design of the study, the data collection, and the analysis, and drafted the manuscript. V.G. conceived of the study, participated in its design and coordination and helped draft the manuscript. All authors contributed to the drafting of the manuscript. All authors have read and approved the final version of the manuscript and agree with the order of presentation of the authors.

## Ethics Statement

Ethical approval for the study was obtained from the Medical Ethics Review Committee of the Amsterdam University Medical Centers.

## Consent

All participants gave their informed consent to take part in the study.

## Conflicts of Interest

The authors declare no conflicts of interest.

## Supporting information


**Data S1:** sms70163‐sup‐0001‐Supinfo1.pdf.

**Data S2:** sms70163‐sup‐0002‐Supinfo2.pdf.

**Data S3:** sms70163‐sup‐0003‐Supinfo3.docx.

**Data S4:** sms70163‐sup‐0004‐Supinfo4.pdf.

**Data S5:** sms70163‐sup‐0005‐Supinfo5.pdf.

## Data Availability

The data that support the findings of this study are available from the corresponding author upon reasonable request.

## References

[1] FIFPRO , Excessive Workload Demands: Player Performance, Recovery and Health (Football Players Worldwide, 2024).

[2] A. Lopez‐Valenciano , I. Ruiz‐Perez , A. Garcia‐Gomez , et al., “Epidemiology of Injuries in Professional Football: A Systematic Review and Meta‐Analysis,” British Journal of Sports Medicine 54, no. 12 (2020): 711–718. 31171515 10.1136/bjsports-2018-099577 PMC9929604

[3] R. M. Page , A. Field , B. Langley , L. D. Harper , and R. Julian , “The Effects of Fixture Congestion on Injury in Professional Male Soccer: A Systematic Review,” Sports Medicine 53, no. 3 (2023): 667–685. 36527592 10.1007/s40279-022-01799-5 PMC9758680

[4] V. Gouttebarge , M. S. Brink , and G. M. M. J. Kerkhoffs , “The Perceptions of Elite Professional Footballers on the International Match Calendar: A Cross‐Sectional Study,” Science and Medicine in Football 3, no. 4 (2019): 339–342.

[5] L. Pillay , D. Burgess , D. C. J. van Rensburg , G. M. Kerkhoffs , and V. Gouttebarge , “The Congested International Match Calendar in Football: Views of 1055 Professional Male Players,” BMC Sports Science, Medicine and Rehabilitation 14, no. 1 (2022): 200. 10.1186/s13102-022-00597-w PMC9706944 36447290

[6] A. B. Bakker and E. Demerouti , “The Job Demands‐Resources Model: State of the Art,” Journal of Managerial Psychology 22, no. 3 (2007): 309–328.

[7] FIFPRO , Player & High Performance Coach Surveys (FIFPRO, 2022).

[8] S. Jünger , S. A. Payne , J. Brine , L. Radbruch , and S. G. Brearley , “Guidance on Conducting and REporting DElphi Studies (CREDES) in Palliative Care: Recommendations Based on a Methodological Systematic Review,” Palliative Medicine 31, no. 8 (2017): 684–706. 28190381 10.1177/0269216317690685

[9] M. I. Yousuf , “The Delphi Technique,” Essays in Education 20, no. 1 (2007): 1–10.

[10] J. McCleery , E. Diamond , R. Kelly , et al., “Centering the Female Athlete Voice in a Sports Science Research Agenda: A Modified Delphi Survey With Team USA Athletes,” British Journal of Sports Medicine 58, no. 19 (2024): 1107–1114. 38981661 10.1136/bjsports-2023-107886 PMC11503037

[11] A. K. Memmini , M. J. Popovich , K. H. Schuyten , et al., “Achieving Consensus Through a Modified Delphi Technique to Create the Post‐Concussion Collegiate Return‐to‐Learn Protocol,” Sports Medicine 53, no. 4 (2022): 903–916. 36396900 10.1007/s40279-022-01788-8 PMC9672536

[12] A. McCall , R. Pruna , N. Van der Horst , et al., “Exercise‐Based Strategies to Prevent Muscle Injury in Male Elite Footballers: An Expert‐Led Delphi Survey of 21 Practitioners Belonging to 18 Teams From the Big‐5 European Leagues,” Sports Medicine 50, no. 9 (2020): 1667–1681. 32676903 10.1007/s40279-020-01315-7 PMC7441050

[13] P. Nasa , R. Jain , and D. Juneja , “Delphi Methodology in Healthcare Research: How to Decide Its Appropriateness,” World Journal of Methodology 11, no. 4 (2021): 116–129. 34322364 10.5662/wjm.v11.i4.116 PMC8299905

[14] I. R. Diamond , R. C. Grant , B. M. Feldman , et al., “Defining Consensus: A Systematic Review Recommends Methodologic Criteria for Reporting of Delphi Studies,” Journal of Clinical Epidemiology 67, no. 4 (2014): 401–409. 24581294 10.1016/j.jclinepi.2013.12.002

[15] H. Bengtsson , J. Ekstrand , and M. Hagglund , “Muscle Injury Rates in Professional Football Increase With Fixture Congestion: An 11‐Year Follow‐Up of the UEFA Champions League Injury Study,” British Journal of Sports Medicine 47, no. 12 (2013): 743–747. 23851296 10.1136/bjsports-2013-092383

[16] H. Bengtsson , J. Ekstrand , M. Walden , and M. Hagglund , “Muscle Injury Rate in Professional Football Is Higher in Matches Played Within 5 Days Since the Previous Match: A 14‐Year Prospective Study With More Than 130 000 Match Observations,” British Journal of Sports Medicine 52, no. 17 (2018): 1116–1122. 29101101 10.1136/bjsports-2016-097399

[17] S. den Hollander , G. Kerkhoffs , and V. Gouttebarge , “The Impact of Match Workload and International Travel on Injuries in Professional Men's Football,” Sports 12, no. 8 (2024): 212. 39195588 10.3390/sports12080212 PMC11360389

[18] A. Dellal , C. Lago‐Peñas , E. Rey , K. Chamari , and E. Orhant , “The Effects of a Congested Fixture Period on Physical Performance, Technical Activity and Injury Rate During Matches in a Professional Soccer Team,” British Journal of Sports Medicine 49, no. 6 (2015): 390–394. 23422422 10.1136/bjsports-2012-091290

[19] G. Dupont , M. Nedelec , A. McCall , D. McCormack , S. Berthoin , and U. Wisloff , “Effect of 2 Soccer Matches in a Week on Physical Performance and Injury Rate,” American Journal of Sports Medicine 38, no. 9 (2010): 1752–1758. 20400751 10.1177/0363546510361236

[20] C. Carling , A. McCall , F. Le Gall , and G. Dupont , “The Impact of Short Periods of Match Congestion on Injury Risk and Patterns in an Elite Football Club,” British Journal of Sports Medicine 50 (2016): 764–768. 26682867 10.1136/bjsports-2015-095501

[21] K. Howle , A. Waterson , and R. Duffield , “Injury Incidence and Workloads During Congested Schedules in Football,” International Journal of Sports Medicine 41, no. 2 (2020): 75–81. 31791088 10.1055/a-1028-7600

[22] B. Delaval , A. E. Abaidia , B. Delecroix , et al., “Recovery During a Congested Schedule and Injury in Professional Football,” International Journal of Sports Physiology and Performance 17, no. 9 (2022): 1399–1406. 35483701 10.1123/ijspp.2021-0504

[23] G. S. Pinheiro , R. C. Quintao , J. G. Claudino , C. Carling , M. Lames , and B. P. Couto , “High Rate of Muscle Injury Despite no Changes in Physical, Physiological and Psychophysiological Parameters in a Professional Football Team During a Long‐Congested Fixture Period,” Research in Sports Medicine 31, no. 6 (2023): 744–755. 35156469 10.1080/15438627.2022.2038159

[24] M. Lackner and H. Sonnabend , “Presenteeism When Employers Are Under Pressure: Evidence From a High‐Stakes Environment,” Economica 90, no. 358 (2023): 477–507.

[25] V. Moreno‐Perez , V. Paredes , D. Pastor , et al., “Under‐Exposure to Official Matches Is Associated With Muscle Injury Incidence in Professional Footballers,” Biology of Sport 38, no. 4 (2021): 563–571. 34937965 10.5114/biolsport.2021.100360 PMC8670809

[26] F. M. Clemente , B. Mendes , P. T. Nikolaidis , F. Calvete , S. Carrico , and A. L. Owen , “Internal Training Load and Its Longitudinal Relationship With Seasonal Player Wellness in Elite Professional Soccer,” Physiology & Behavior 179 (2017): 262–267. 28668619 10.1016/j.physbeh.2017.06.021

[27] K. Howle , A. Waterson , and R. Duffield , “Recovery Profiles Following Single and Multiple Matches Per Week in Professional Football,” European Journal of Sport Science 19, no. 10 (2019): 1303–1311. 30998434 10.1080/17461391.2019.1601260

[28] A. Garcia‐Romero‐Perez , F. J. Ordonez , F. Reyes‐Gil , E. S. Rodriguez‐Lopez , and A. Oliva‐Pascual‐Vaca , “Muscle Damage Biomarkers in Congestion Weeks in English Premier League Soccer Players: A Prospective Study for Two Consecutive Seasons,” International Journal of Environmental Research and Public Health 18, no. 15 (2021): 7960. 34360252 10.3390/ijerph18157960 PMC8345565

[29] K. Saidi , H. Zouhal , F. Rhibi , et al., “Effects of a Six‐Week Period of Congested Match Play on Plasma Volume Variations, Hematological Parameters, Training Workload and Physical Fitness in Elite Soccer Players,” PLoS One 14, no. 7 (2019): e0219692. 31344056 10.1371/journal.pone.0219692 PMC6657839

[30] D. Noor , A. McCall , M. Jones , et al., “Perceived Load, Fatigue and Recovery Responses During Congested and Non‐Congested Micro‐Cycles in International Football Tournaments,” Journal of Science and Medicine in Sport 24, no. 12 (2021): 1278–1283. 34452841 10.1016/j.jsams.2021.07.001

[31] T. R. Lundberg and K. Weckstrom , “Fixture Congestion Modulates Post‐Match Recovery Kinetics in Professional Soccer Players,” Research in Sports Medicine 25, no. 4 (2017): 408–420. 28795586 10.1080/15438627.2017.1365296

[32] K. Saidi , A. Ben Abderrahman , D. Boullosa , et al., “The Interplay Between Plasma Hormonal Concentrations, Physical Fitness, Workload and Mood State Changes to Periods of Congested Match Play in Professional Soccer Players,” Frontiers in Physiology 11 (2020): 835. 32792977 10.3389/fphys.2020.00835 PMC7385323

[33] R. Morgans , D. Adams , R. Mullen , C. Mclellan , and M. Williams , “An English Championship League Soccer Team Did Not Experience Impaired Physical Match Performance in a Second Match After 75 Hours Recovery,” Journal of Australian Strength and Conditioning 22, no. 4 (2014): 16–23.

[34] K. Saidi , H. Zouhal , D. Boullosa , et al., “Biochemical Markers and Wellness Status During a Congested Match Play Period in Elite Soccer Players,” International Journal of Sports Physiology and Performance 17, no. 4 (2022): 605–620. 35038677 10.1123/ijspp.2020-0914

[35] J. Ekstrand , M. Waldén , and M. Hägglund , “A Congested Football Calendar and the Wellbeing of Players: Correlation Between Match Exposure of European Footballers Before the World Cup 2002 and Their Injuries and Performances During That World Cup,” British Journal of Sports Medicine 38, no. 4 (2004): 493–497. 15273193 10.1136/bjsm.2003.009134 PMC1724854

[36] C. Lago‐Penas , E. Rey , J. Lago‐Ballesteros , L. Casais , and E. Dominguez , “The Influence of a Congested Calendar on Physical Performance in Elite Soccer,” Journal of Strength and Conditioning Research 25, no. 8 (2011): 2111–2117. 21572352 10.1519/JSC.0b013e3181eccdd2

[37] C. Lago‐Penas , “Consequences of a Busy Soccer Match Schedule on Team Performance: Empirical Evidence From Spain,” International Sportmed Journal 10, no. 2 (2009): 86–94.

[38] E. Rey , C. Lago‐Penas , J. Lago‐Ballesteros , L. Casais , and A. Dellal , “The Effect of a Congested Fixture Period on the Activity,” Biology of Sport 27 (2010): 181–185.

[39] R. Silva , H. I. Ceylan , G. Badicu , et al., “Match‐To‐Match Variations in External Load Measures During Congested Weeks in Professional Male Soccer Players,” Journal of Men's Health 17, no. 4 (2021): 207–217.

[40] C. Carling , F. L. Gall , and T. P. Reilly , “Effects of Physical Efforts on Injury in Elite Soccer,” International Journal of Sports Medicine 31, no. 3 (2010): 180–185. 20024885 10.1055/s-0029-1241212

[41] R. Morgans , P. Orme , L. Anderson , B. Drust , and J. P. Morton , “An Intensive Winter Fixture Schedule Induces a Transient Fall in Salivary IgA in English Premier League Soccer Players,” Research in Sports Medicine 22, no. 4 (2014): 346–354. 25295473 10.1080/15438627.2014.944641

[42] C. Carling , F. Le Gall , and G. Dupont , “Are Physical Performance and Injury Risk in a Professional Soccer Team in Match‐Play Affected Over a Prolonged Period of Fixture Congestion?,” International Journal of Sports Medicine 33, no. 1 (2012): 36–42. 22012641 10.1055/s-0031-1283190

[43] M. Waldén , M. Hägglund , and J. Ekstrand , “UEFA Champions League Study: A Prospective Study of Injuries in Professional Football During the 2001–2002 Season,” British Journal of Sports Medicine 39, no. 8 (2005): 542–546. 16046340 10.1136/bjsm.2004.014571 PMC1725291

[44] C. W. Fuller , “Modeling the Impact of Players' Workload on the Injury‐Burden of English Premier League Football Clubs,” Scandinavian Journal of Medicine & Science in Sports 28, no. 6 (2018): 1715–1721. 29474738 10.1111/sms.13078

[45] T. Dalen‐Lorentsen , T. E. Andersen , C. Thorbjornsen , et al., “Injury Characteristics in Norwegian Male Professional Football: A Comparison Between a Regular Season and a Season in the Pandemic,” Frontiers in Sports and Active Living 4 (2022): 915581. 36339642 10.3389/fspor.2022.915581 PMC9635315

[46] E. Penedo‐Jamardo , E. Rey , A. Padrón‐Cabo , and A. Kalén , “The Impact of Different Recovery Times Between Matches on Physical and Technical Performance According to Playing Positions,” International Journal of Performance Analysis in Sport 17, no. 3 (2017): 271–282.

[47] A. Ascensao , A. Rebelo , E. Oliveira , F. Marques , L. Pereira , and J. Magalhaes , “Biochemical Impact of a Soccer Match ‐ Analysis of Oxidative Stress and Muscle Damage Markers Throughout Recovery,” Clinical Biochemistry 41, no. 10–11 (2008): 841–851. 18457670 10.1016/j.clinbiochem.2008.04.008

[48] P. Krustrup , N. Ørtenblad , J. Nielsen , et al., “Maximal Voluntary Contraction Force, SR Function and Glycogen Resynthesis During the First 72 h After a High‐Level Competitive Soccer Game,” European Journal of Applied Physiology 111, no. 12 (2011): 2987–2995. 21448723 10.1007/s00421-011-1919-y

[49] E. Rampinini , A. Bosio , I. Ferraresi , A. Petruolo , A. Morelli , and A. Sassi , “Match‐Related Fatigue in Soccer Players,” Medicine & Science in Sports & Exercise 43, no. 11 (2011): 2161–2170. 21502891 10.1249/MSS.0b013e31821e9c5c

[50] K. Funten , O. Faude , J. Lensch , and T. Meyer , “Injury Characteristics in the German Professional Male Soccer Leagues After a Shortened Winter Break,” Journal of Athletic Training 49, no. 6 (2014): 786–793. 25365132 10.4085/1062-6050-49.3.51 PMC4264651

[51] J. Ekstrand , A. Spreco , and M. Davison , “Elite Football Teams That Do Not Have a Winter Break Lose on Average 303 Player‐Days More Per Season to Injuries Than Those Teams That Do: A Comparison Among 35 Professional European Teams,” British Journal of Sports Medicine 53, no. 19 (2019): 1231–1235. 30442720 10.1136/bjsports-2018-099506

[52] M. Jamil , S. A. McErlain‐Naylor , and M. Beato , “Investigating the Impact of the Mid‐Season Winter Break on Technical Performance Levels Across European Football—Does a Break in Play Affect Team Momentum?,” International Journal of Performance Analysis in Sport 20, no. 3 (2020): 406–419.

[53] A. Rodriguez‐Fernandez , J. Sanchez‐Sanchez , R. Ramirez‐Campillo , J. A. Rodriguez‐Marroyo , J. G. Villa Vicente , and F. Y. Nakamura , “Effects of Short‐Term In‐Season Break Detraining on Repeated‐Sprint Ability and Intermittent Endurance According to Initial Performance of Soccer Player,” PLoS One 13, no. 8 (2018): e0201111. 30110374 10.1371/journal.pone.0201111 PMC6093601

[54] P. M. Fowler , A. McCall , M. Jones , and R. Duffield , “Effects of Long‐Haul Transmeridian Travel on Player Preparedness: Case Study of a National Team at the 2014 FIFA World Cup,” Journal of Science and Medicine in Sport 20, no. 4 (2017): 322–327. 28109712 10.1016/j.jsams.2016.08.021

[55] E. Clements , F. Ehrmann , A. Clark , M. Jones , A. McCall , and R. Duffield , “Flight Path and Scheduling Effects on Perceived Jet Lag, Fatigue, and Sleep in Footballers Traveling to and From National Teams,” International Journal of Sports Physiology and Performance 18, no. 10 (2023): 1132–1140. 37369367 10.1123/ijspp.2023-0027

[56] E. Clements , F. Ehrmann , A. Clark , M. Jones , D. Lu , and R. Duffield , “The Type and Extent of Travel for Professional Footballers Undertaking National Team Duties for a National Football Federation,” Biology of Sport 40, no. 3 (2023): 707–713. 37398973 10.5114/biolsport.2023.119288 PMC10286598

[57] M. Lastella , G. D. Roach , and C. Sargent , “Travel Fatigue and Sleep/Wake Behaviors of Professional Soccer Players During International Competition,” Sleep Health 5, no. 2 (2019): 141–147. 30928113 10.1016/j.sleh.2018.10.013

[58] E. J. Gilbert , J. C. Dixon , and T. M. Loughead , “An Examination of Travel Effects on Performance Outcomesin Major League Soccer,” Journal of Applied Sport Management 12, no. 1 (2020): 4.

[59] M. Zacharko , M. Konefal , L. Radziminski , et al., “Direction of Travel of Time Zones Crossed and Results Achieved by Soccer Players. The Road From the 2018 FIFA World Cup to UEFA EURO 2020,” Research in Sports Medicine 30, no. 2 (2022): 145–155. 33251863 10.1080/15438627.2020.1853545

[60] H. H. Fullagar , R. Duffield , S. Skorski , et al., “Sleep, Travel, and Recovery Responses of National Footballers During and After Long‐Haul International Air Travel,” International Journal of Sports Physiology and Performance 11, no. 1 (2016): 86–95. 25946072 10.1123/ijspp.2015-0012

[61] P. Fowler , R. Duffield , K. Howle , A. Waterson , and J. Vaile , “Effects of Northbound Long‐Haul International Air Travel on Sleep Quantity and Subjective Jet Lag and Wellness in Professional Australian Soccer Players,” International Journal of Sports Physiology and Performance 10, no. 5 (2015): 648–654. 25569181 10.1123/ijspp.2014-0490

[62] E. Clements , F. Ehrmann , A. Clark , M. Jones , A. McCall , and R. Duffield , “Travel Across More Time Zones Results in Worse Perceived Fatigue and Sleep in National‐Team Footballers,” International Journal of Sports Physiology and Performance 18, no. 3 (2023): 268–275. 36716744 10.1123/ijspp.2022-0230

[63] R. F. Liporaci , S. Yoshimura , and B. M. Baroni , “Perceptions of Professional Football Players on Injury Risk Factors and Prevention Strategies,” Science and Medicine in Football 6, no. 2 (2021): 148–152. 35475749 10.1080/24733938.2021.1937689

[64] A. Field , L. D. Harper , B. C. R. Chrismas , et al., “The Use of Recovery Strategies in Professional Soccer: A Worldwide Survey,” International Journal of Sports Physiology and Performance 16, no. 12 (2021): 1804–1815. 34051698 10.1123/ijspp.2020-0799

[65] A. McCall , C. Carling , M. Nedelec , et al., “Risk Factors, Testing and Preventative Strategies for Non‐Contact Injuries in Professional Football: Current Perceptions and Practices of 44 Teams From Various Premier Leagues,” British Journal of Sports Medicine 48, no. 18 (2014): 1352–1357. 24837243 10.1136/bjsports-2014-093439

[66] A. McCall , G. Dupont , and J. Ekstrand , “Injury Prevention Strategies, Coach Compliance and Player Adherence of 33 of the UEFA Elite Club Injury Study Teams: A Survey of Teams' Head Medical Officers,” British Journal of Sports Medicine 50, no. 12 (2016): 725–730. 26795611 10.1136/bjsports-2015-095259

[67] V. Di Salvo , D. Bonanno , M. Modonutti , et al., “Perspectives on Postmatch Fatigue From 300 Elite European Soccer Players,” International Journal of Sports Physiology and Performance 18, no. 1 (2023): 55–60. 36521189 10.1123/ijspp.2022-0200

[68] International Labour Organization , Rest Periods: Definitions and Dimensions (ILO, 2015).

[69] FIFPRO , Extreme Calendar Congestion: The Adverse Effects on Player Health and Wellness (FIFPRO, 2023).

[70] M.‐J. Wu , K. Zhao , and F. Fils‐Aime , “Response Rates of Online Surveys in Published Research: A Meta‐Analysis,” Computers in Human Behavior Reports 7 (2022): 100206.

[71] T. M. Sabato , T. J. Walch , and D. J. Caine , “The Elite Young Athlete: Strategies to Ensure Physical and Emotional Health,” Open Access Journal of Sports Medicine 7 (2016): 99–113. 27621677 10.2147/OAJSM.S96821 PMC5012846

