# Supplementary material for: The International Match Calendar in Men's Professional Football: An Expert Position Statement
Source: Scand J Med Sci Sports. 2025 Nov 6;35(11):e70163. doi: 10.1111/sms.70163 (PMC12591028; doi:10.1111/sms.70163)
Supplement: Supplementary file 1 — Data S1: sms70163‐sup‐0001‐Supinfo1.pdf. [file SMS-35-e70163-s003.pdf]

Supplemental file A

Search strategy Medline

#1: Soccer[Mesh] OR soccer[tiab] OR football*[tiab]

#2: “match calendar”[tiab] OR “match congestion”[tiab] OR “fixture congestion”[tiab] OR recovery[tiab] OR rest[tiab] OR break[tiab] OR regenerat*[tiab] OR “match workload”[tiab] OR travel[tiab]

3: #1 AND #2

Search strategy SPORTDiscus

S1: TI(soccer OR football*) OR AB(soccer OR football*)

S2: TI(“match calendar” OR “match congestion” OR “fixture congestion” OR recovery OR rest OR break OR regenerat* OR “match workload” OR travel) OR AB(“match calendar” OR “match congestion” OR “fixture congestion” OR recovery OR rest OR break OR regenerat* OR “match workload” OR travel)

S3: S1 AND S2
