# Supplementary material for: The International Match Calendar in Men's Professional Football: An Expert Position Statement
Source: Scand J Med Sci Sports. 2025 Nov 6;35(11):e70163. doi: 10.1111/sms.70163 (PMC12591028; doi:10.1111/sms.70163)
Supplement: Supplementary file 2 — Data S2: sms70163‐sup‐0002‐Supinfo2.pdf. [file SMS-35-e70163-s005.pdf]

Supplemental file B

KU LEUVEN

Impact of Workload on Football Players' Health and Wellbeing: Medical and Legal Perspectives

Lode Godderis[1,2*], Kelly Mortelmans[2], Frank Hendrickx[3]

1: KU Leuven, Faculty of Medicine, Department of Primary Care and Public Health, Leuven, Belgium.

2: Idewe, External Service for Prevention and Protection at Work, Heverlee, Belgium

3: KU Leuven, Faculty of Law and Criminology; head of the Institute for Labour Law, Leuven, Belgium.

* Contact information: Prof dr Lode Godderis: lode.godderis@kuleuven.be

Table of Contents

Introduction
Analysis of the professional soccer player occupational health
- Introduction
- Methods
- Results
- Occupational health perspective
- Recommendations
- Conclusions

Occupational health and safety standards for the football industry: a legal analysis
- Introduction
- Labour standards and professional football
- OSH as a fundamental human right
- International and European OSH standards
- Tailoring OSH to professional football
- Conclusions

Key findings

Acknowledgements

This research was supported by funding from FIFPRO.

We would like to thank Drs Fleur Van Lint, medical intern, for her valuable assistance with the literature search and analysis.

Introduction

Increasing demands placed on professional soccer players have raised significant concern among players, coaches, and sports health professionals regarding their health, wellbeing, and overall workload management. The impact of overloaded schedules is felt not only physically but also mentally, creating a challenging environment for professional soccer players. The FiFPRO Player Workload Monitoring Platform has monitored player workload for several years. The 2022 FIFPRO Player Workload Monitoring report, which includes data from over 1,000 players across major European leagues, underscores these challenges. An alarming 55 percent of players reported injuries due to overloaded schedules, while 40 percent indicated that a congested calendar adversely affected their mental health. Furthermore, 50 percent noted that their off-seasons were cut short by their clubs or national teams.[1] These findings are supported by high-performance experts—including sports scientists, doctors, and conditioning coaches—who warn that the current match volume, without adequate protective regulations, poses significant risks to players' mental and physical health. These risks are exacerbated by the unique occupational environment of professional soccer, which is characterized by high physical demands, frequent travel, intense competition and an increasingly expanding match schedule, reflecting a rise in the number of fixtures. In this context, injuries, not only physical but also psychological stress and burnout, should be viewed as occupational injuries requiring systematic management and preventive strategies aligned with Occupational Safety and Health (OSH) principles. Unlike other professions, athletes often face a greater acceptance of occupational risks, driven by the relentless pressure to succeed and a "win at all costs" mentality. This competitive drive can lead to behaviors that compromise both health and safety. By enhancing OSH awareness and prioritizing health and safety over short-term performance goals, more conservative and safety-oriented decision-making processes can be promoted.[2]

The first objective of this project is to examine the consequences of the excessive workload on players (in particular in terms of their risk of injury, their performance, their mental health, their careers and their privacy and family life). The second objective of this project is to investigate whether the legislation related to the occupational safety and health can provide a sufficient legal basis to safeguard a holistic approach to football players' health, considering the risks for their physical and mental workload due to, amongst others, a congested football calendar.

---

[1] 2022/23 Men's Workload Report: Extreme Calendar Congestion; https://fifpro.org/en

[2] Chen Y, Buggy C, Kelly S. Winning at all costs: a review of risk-taking behaviour and sporting injury from an occupational safety and health perspective. Sports Med Open. 2019 May 2;5(1):15. doi: 10.1186/s40798-019-0189-9. PMID: 31049736; PMCID: PMC6497707.

Analysis of the professional soccer player occupational health

Prof Dr Lode Godderis, Dr Kelly Mortelmans, Drs Fleur Van Lint

Introduction

Concerns about the increasing workload of professional soccer players have been growing among players, coaches, and health professionals. The physical intensity of training sessions, combined with a congested match schedule, presents significant challenges not only to players' physical health but also to their mental well-being. However, these demands are not limited to the pitch alone. Professional soccer players also face a range of additional stressors. To effectively address these multifaceted concerns, it is essential to comprehensively map out all elements contributing to player workload. This involves understanding not just the direct demands of training and matches, but also the less apparent but equally impactful factors that can lead to physical strain and psychological stress. By conducting a thorough literature review, we aim to identify these various determinants and translate them into an occupational health framework, using the Job Demands-Resources (JDR) model[3]. The JDR model provides a structured approach to analyze how job demands interact with job resources. Applying this model to the context of professional soccer allows for a nuanced understanding of the balance between demands and resources. This approach will enable us to develop comprehensive strategies to better manage player health, mitigate risks of burnout and injury, and ultimately promote a sustainable and balanced approach to their careers.

Methods

To explore the demands, resources, and potential health consequences for professional soccer players, we conducted a systematic literature review. Acknowledging the need for a comprehensive approach, our study examines all aspects of professional soccer players as employees, including their physical, mental, and psychological well-being. An electronic search was conducted in PubMed and Scopus for research published before June 30, 2024. We used different search-strings to identify relevant studies. Study identification and selection were independently performed by two researchers. The process involved saving search results, screening titles and abstracts, removing duplicates, and reviewing full texts. Initially, 360 articles were retrieved (263 from the first search string and 97 from the second). After screening titles and abstracts, 217 articles were excluded due to irrelevance, and duplicates were also removed. This left 69 articles for full-text screening. Ultimately, 37 articles met the eligibility criteria and were included in the review. To be included in the study, articles had to meet several criteria: they had to be written in English, categorized as a review, systematic review, or meta-analysis, and focus on professional soccer players, providing data on demands, resources, or potential negative health outcomes. In cases where a mixed population of professional, semi-professional, or amateur players was involved, data were included only if relevant to professional players. Data extraction was

---

[3] Bakker, A.B. and Demerouti, E. (2007), "The Job Demands-Resources model: state of the art", *Journal of Managerial Psychology*, Vol. 22 No. 3, pp. 309-328. https://doi.org/10.1108/02683940710733115

conducted systematically. Extracted data were categorized to ensure comprehensive analysis, covering general study descriptors, study aims, key findings, and relevance to the JD-R model. Potential negative health outcomes were documented.

Results

After thoroughly reviewing the included papers, six predominant themes emerged: fatigue, injury, mental health, load, monitoring, and lifestyle factors. This division is not absolute, as various themes were addressed across multiple articles .

1. Fatigue

Fatigue is described as a combination of dehydration, glycogen depletion, muscle damage, and mental fatigue[4]-[5]. The importance of recovery strategies, such as proper hydration, diet[6], and sleep[7], is emphasized, alongside the need for adequate recovery time. The International Olympic Committee recommends that soccer matches should be spaced at least 96 hours apart to protect players from injury and ensure sufficient recovery[8].

2. Injury

Football has a higher incidence rate of injuries than traditional industrial occupations[9], with 36 injuries per 1000 match-hours and 3.7 injuries per 1000 training-hours[10]-[11]. Several factors contribute to this high rate, including the increasingly intense nature of the game[5], more frequent high-intensity training sessions, previous injuries[12]-[13], advancing age, a growing number of matches and

---

[4] González-Víllora, Sixto & Prieto Ayuso, Alejandro & Cardoso, Felippe & Teoldo da Costa, Israel. (2022). The role of mental fatigue in soccer: a systematic review. International Journal of Sports Science & Coaching. 1-14. 10.1177/17479541211069536.

[5] Nédélec M, Halson S, Abaidia AE, Ahmaidi S, Dupont G. Stress, Sleep and Recovery in Elite Soccer: A Critical Review of the Literature. Sports Med. 2015 Oct;45(10):1387-400. doi: 10.1007/s40279-015-0358-z. PMID: 26206724.

[6] Ranchordas MK, Dawson JT, Russell M. Practical nutritional recovery strategies for elite soccer players when limited time separates repeated matches. J Int Soc Sports Nutr. 2017 Sep 12;14:35. doi: 10.1186/s12970-017-0193-8. PMID: 28919844; PMCID: PMC5596842.

[7] Nédélec M, Halson S, Delecroix B, Abaidia AE, Ahmaidi S, Dupont G. Sleep Hygiene and Recovery Strategies in Elite Soccer Players. Sports Med. 2015 Nov;45(11):1547-59. doi: 10.1007/s40279-015-0377-9. PMID: 26275673.

[8] Silva JR, Rumpf MC, Hertzog M, Castagna C, Farooq A, Girard O, Hader K. Acute and Residual Soccer Match-Related Fatigue: A Systematic Review and Meta-analysis. Sports Med. 2018 Mar;48(3):539-583. doi: 10.1007/s40279-017-0798-8. PMID: 29098658.

[9] Klein, Christian & Henke, Thomas & Platen, Petra. (2018). Injuries in football (soccer)—a systematic review of epidemiology and aetiological aspects. German Journal of Exercise and Sport Research. 48. 10.1007/s12662-018-0530-3.

[10] Sanmiguel-Rodríguez, Alberto. (2021). Injuries in High-Performance Football: A Systematic Review. Sport Mont Journal. 19. 107-114. 10.26773/smj.211009.

[11] Cardoso-Marinho B, Barbosa A, Bolling C, Marques JP, Figueiredo P, Brito J. The perception of injury risk and prevention among football players: A systematic review. Front Sports Act Living. 2022 Dec 7;4:1018752. doi: 10.3389/fspor.2022.1018752. PMID: 36570494; PMCID: PMC9768495.

[12] Nassis GP, Verhagen E, Brito J, Figueiredo P, Krustrup P. A review of machine learning applications in soccer with an emphasis on injury risk. Biol Sport. 2023 Jan;40(1):233-239. doi:10.5114/biolsport.2023.114283. Epub 2022 Mar 16. PMID: 36636180; PMCID: PMC9806760.

[13] Slimani M, Bragazzi NL, Znazen H, Paravlic A, Azaiez F, Tod D. Psychosocial predictors and psychological prevention of soccer injuries: A systematic review and meta-analysis of the literature. Phys Ther Sport. 2018 Jul;32:293-300. doi: 10.1016/j.ptsp.2018.05.006. Epub 2018 Jun 20. PMID: 29776844.

shorter recovery periods[8-14-15-16-17-18-19-20-21-22-23-24]. Effective load management, sufficient recovery time, and injury prevention programs[25] are identified as protective factors that can help reduce the risk of injuries and promote overall player health and performance.

3. Mental Health

Depressive symptoms are more common among football players than in the general population[22]. Increased training loads, combined with insufficient recovery, can negatively impact players' moods and overall mental health[26]. Psychosocial factors, such as a history of stressors and certain personality traits, are consistent predictors of injury rates. Integrating psychological interventions and regular mood monitoring into injury prevention programs can enhance players' mental health and performance, ultimately reducing injury rates and promoting overall well-being, particularly in managing the challenges of constant travel and game-related pressures.[27]

---

[14] Clemente FM, Afonso J, Costa J, Oliveira R, Pino-Ortega J, Rico-González M. Relationships between Sleep, Athletic and Match Performance, Training Load, and Injuries: A Systematic Review of Soccer Players. Healthcare (Basel). 2021 Jun 26;9(7):808. doi: 10.3390/healthcare9070808. PMID: 34206948; PMCID: PMC8305909.

[15] Hughes T, Sergeant JC, Parkes MJ, *et al* Prognostic factors for specific lower extremity and spinal musculoskeletal injuries identified through medical screening and training load monitoring in professional football (soccer): a systematic review *BMJ Open Sport & Exercise Medicine* 2017;**3:**e000263. doi: 10.1136/bmjsem-2017-000263

[16] Jaspers A, Brink MS, Probst SG, Frencken WG, Helsen WF. Relationships Between Training Load Indicators and Training Outcomes in Professional Soccer. Sports Med. 2017 Mar;47(3):533-544. doi: 10.1007/s40279-016-0591-0. PMID: 27459866.

[17] Jiang Z, Hao Y, Jin N, Li Y. A Systematic Review of the Relationship between Workload and Injury Risk of Professional Male Soccer Players. Int J Environ Res Public Health. 2022 Oct 14;19(20):13237. doi: 10.3390/ijerph192013237. PMID: 36293817; PMCID: PMC9602492.

[18] Nédélec M, McCall A, Carling C, Legall F, Berthoin S, Dupont G. Recovery in soccer: part I - post-match fatigue and time course of recovery. Sports Med. 2012 Dec 1;42(12):997-1015. doi: 10.2165/11635270-000000000-00000. PMID: 23046224.

[19] Page RM, Field A, Langley B, Harper LD, Julian R. The Effects of Fixture Congestion on Injury in Professional Male Soccer: A Systematic Review. Sports Med. 2023 Mar;53(3):667-685. doi: 10.1007/s40279-022-01799-5. Epub 2022 Dec 17. PMID: 36527592; PMCID: PMC9758680.

[20] Pérez-Castillo ÍM, Rueda R, Bouzamondo H, López-Chicharro J, Mihic N. Biomarkers of post-match recovery in semi-professional and professional football (soccer). Front Physiol. 2023 Apr 11;14:1167449. doi: 10.3389/fphys.2023.1167449. PMID: 37113691; PMCID: PMC10126523.

[21] Rago V, Brito J, Figueiredo P, Costa J, Krustrup P, Rebelo A. Internal training load monitoring in professional football: a systematic review of methods using rating of perceived exertion. J Sports Med Phys Fitness. 2020 Jan;60(1):160-171. doi: 10.23736/S0022-4707.19.10000-X. Epub 2019 Oct 24. PMID: 31663318.

[22] Sarmento H, Frontini R, Marques A, Peralta M, Ordoñez-Saavedra N, Duarte JP, Figueiredo A, Campos MJ, Clemente FM. Depressive Symptoms and Burnout in Football Players: A Systematic Review. Brain Sci. 2021 Oct 14;11(10):1351. doi: 10.3390/brainsci11101351. PMID: 34679415; PMCID: PMC8534279.

[23] Teixeira JE, Forte P, Ferraz R, Leal M, Ribeiro J, Silva AJ, Barbosa TM, Monteiro AM. Monitoring Accumulated Training and Match Load in Football: A Systematic Review. Int J Environ Res Public Health. 2021 Apr 8;18(8):3906. doi: 10.3390/ijerph18083906. PMID: 33917802; PMCID: PMC8068156.

[24] Springham M, Newton RU, Strudwick AJ, Waldron M. Selected Immunoendocrine Measures for Monitoring Responses to Training and Match Load in Professional Association Football: A Review of the Evidence. Int J Sports Physiol Perform. 2022 Nov 7;17(12):1654-1663. doi: 10.1123/ijspp.2022-0226. PMID: 36343621.

[25] Al Attar WSA, Soomro N, Sinclair PJ, Pappas E, Sanders RH. Effect of Injury Prevention Programs that Include the Nordic Hamstring Exercise on Hamstring Injury Rates in Soccer Players: A Systematic Review and Meta-Analysis. Sports Med. 2017 May;47(5):907-916. doi: 10.1007/s40279-016-0638-2. PMID: 27752982.

[26] Selmi O, Ouergui I, Muscella A, My G, Marsigliante S, Nobari H, Suzuki K, Bouassida A. Monitoring Psychometric States of Recovery to Improve Performance in Soccer Players: A Brief Review. Int J Environ Res Public Health. 2022 Jul 31;19(15):9385. doi: 10.3390/ijerph19159385. PMID: 35954741; PMCID: PMC9367927.

[27] Selmi O, Ouergui I, Muscella A, Levitt DE, Suzuki K, Bouassida A. Monitoring mood state to improve performance in soccer players: A brief review. Front Psychol. 2023 Feb 22;14:1095238. doi: 10.3389/fpsyg.2023.1095238. PMID: 36910823; PMCID: PMC9992435.

4. Load Management

Variations in training and match loads in football are influenced by several factors, including weekly schedules, player status, position, age group, training mode, and contextual elements. Although detailed indicators of training load are available, their effectiveness in predicting training outcomes remains unclear.[23]

5. Monitoring

Current methods for injury prediction through screening and monitoring are limited. Different types of monitoring are used, including physical load monitoring, psychological monitoring, and biomarker monitoring. However, the effectiveness of these methods in predicting injuries and optimizing training outcomes remains uncertain.[28]

6. Lifestyle Factors

Various factors contribute to sleep desynchronization among soccer players, including late-night matches, exposure to bright light, consumption of caffeine or alcohol, travel fatigue, inconsistent match schedules, and individual variability in sleep requirements.[7-14] Given the high demands on professional soccer players, nutritional recovery is essential for both performance and injury prevention. Implementing effective sleep and nutritional strategies can reduce injury risk.[6-8]

Occupational health perspective

The articles were re-examined through an occupational health lens to identify potential job demands, resources, health outcomes, and other factors that align with the Job Demands-Resources (JDR) model, in order to obtain a comprehensive and complete understanding of the various elements affecting professional soccer players. This analysis is illustrated in Figure 1.

Translating performance and sports literature into an occupational health model reveals several key demands that are frequently highlighted in current research, including physical workload, match congestion, and fatigue. These elements are well-documented and are widely acknowledged as significant challenges for professional soccer players, potentially leading to injuries, reduced performance, and long-term health issues. However, this translation also exposes gaps in the literature where other aspects have not been explored with the same depth or frequency. By incorporating insights from occupational health literature, we can supplement these findings and provide a more comprehensive view of the factors influencing player well-being.

Several articles mention that travel increases the workload of soccer players. To better understand this, we can refer to other literature that highlights two key conditions: travel fatigue and jet lag. Travel fatigue develops gradually over the course of any long journey, regardless of the mode of transport, and includes symptoms such as general fatigue and disorientation. Jet lag, on the other hand, is a sleep disorder that occurs after trans-meridian travel. Long-haul travel also poses

[28] Djaoui L, Haddad M, Chamari K, Dellal A. Monitoring training load and fatigue in soccer players with physiological markers. Physiol Behav. 2017 Nov 1;181:86-94. doi: 10.1016/j.physbeh.2017.09.004. Epub 2017 Sep 5. Erratum in: Physiol Behav. 2018 Oct 1;194:589. doi: 10.1016/j.physbeh.2018.02.041. PMID: 28886966.

additional risks, including an increased risk of venous thromboembolism, upper respiratory tract infections, and gastrointestinal symptoms.[29-30]

Another challenging element is the fluctuating match times, particularly late-evening games. Players are expected to perform at their peak during hours when their circadian rhythm naturally promotes sleepiness. Taking this into account, we can draw a comparison between soccer players and night shift workers. Worker fatigue, extensively studied in shift workers, is associated with several adverse health outcomes, including cardiovascular disease and metabolic syndrome. The lack of sufficient rest also impairs cognitive function.[31] For soccer players, this means that cumulative fatigue from night matches could potentially compromise their on-field performance, increase the risk of injuries, and negatively affect their overall career longevity.

Team culture also plays an important role in promoting both mental health and physical safety. A culture that prioritizes health and safety fosters an environment where players are supported not just in their athletic endeavors but in their overall well-being. This mirrors the occupational health concept, where a workplace that values health and safety tends to have better employee outcomes, including lower rates of injury and higher job satisfaction.

By applying the JDR model, we can gain a clearer understanding of the traits specific to different player profiles. This approach helps in identifying additional stressors and potential interventions, enabling to develop a more tailored and effective support system. High profile players operate at various levels of competition, not only representing their club teams but also their national teams. This dual involvement introduces a unique set of dynamics to their overall workload. Beyond the increased number of matches, players face additional demands such as extensive travel schedules, heightened media attention and the pressure of balancing multiple roles.

Adolescent professional soccer players experience a set of distinctive challenges, particularly due to their different developmental stages, which require specific load patterns. Their training responses vary from adult players as their sport-specific skills and physical characteristics are still maturing. In addition to the physical demands of the sport, they also experience stressors such as academic pressures and age-related expectations.[32]

---

[29] Doherty R, Madigan SM, Nevill A, Warrington G, Ellis JG. The impact of long haul travel on the sleep of elite athletes. Neurobiol Sleep Circadian Rhythms. 2023 Sep 20;15:100102. doi: 10.1016/j.nbscr.2023.100102. PMID: 37766939; PMCID: PMC10520441.

[30] Rossiter A, Warrington GD, Comyns TM. Effects of Long-Haul Travel on Recovery and Performance in Elite Athletes: A Systematic Review. J Strength Cond Res. 2022 Nov 1;36(11):3234-3245. doi: 10.1519/JSC.0000000000004021. Epub 2021 Mar 18. PMID: 36287181.

[31] Garde AH, Begtrup L, Bjorvatn B, Bonde JP, Hansen J, Hansen ÅM, Härmä M, Jensen MA, Kecklund G, Kolstad HA, Larsen AD, Lie JA, Moreno CR, Nabe-Nielsen K, Sallinen M. How to schedule night shift work in order to reduce health and safety risks. Scand J Work Environ Health. 2020 Nov 1;46(6):557-569. doi: 10.5271/sjweh.3920. Epub 2020 Sep 8. PMID: 32895725; PMCID: PMC7737811.

[32] Verstappen S, van Rijn RM, Cost R, Stubbe JH. The Association Between Training Load and Injury Risk in Elite Youth Soccer Players: a Systematic Review and Best Evidence Synthesis. Sports Med Open. 2021 Jan 11;7(1):6. doi: 10.1186/s40798-020-00296-1. PMID: 33428001; PMCID: PMC7801562.

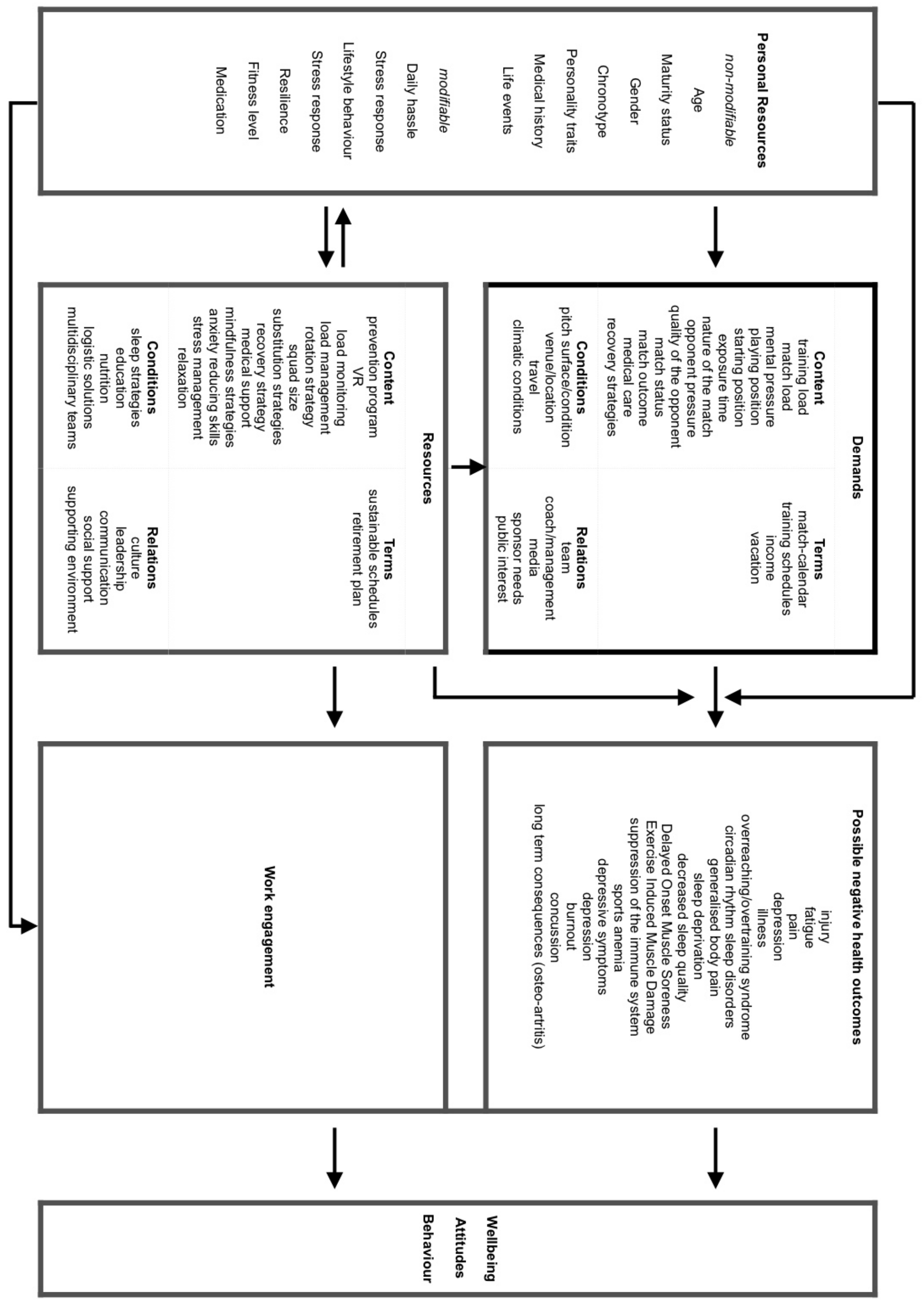

Figure 1. Key determinants workload based on the conducted systematic literature search and applied to the JDR model.

Recommendations

There already is a significant focus on monitoring the health status of players, and various interventions are being implemented to manage or mitigate their workload. However, a more structured, holistic approach is needed—one that systematically identifies and assesses all potential risks and determinants and aligns them with targeted actions grounded in occupational health principles. This can be achieved by performing comprehensive risk assessments that take into account all dimensions of the work environment.

1. Risk Assessments

These assessments should cover all domains of work, including job content (the physical and mental demands of playing and training), work relations (the quality of interactions with teammates, coaches, and support staff), employment terms (contract conditions, job security, and remuneration), and working conditions (such as travel schedules and facilities). A holistic risk assessment must also incorporate medical, ergonomic, psychosocial, and occupational hygiene factors. Medically, it is important to monitor physical health markers such as injury rates and recovery times. Ergonomically, the design of training practices and equipment needs to be optimized to prevent overuse injuries and ensure physical safety. Psychosocially, attention should be given to mental health stressors, such as performance pressure, media scrutiny, and team dynamics, which can contribute to burnout and other psychological issues. Occupational hygiene factors, like exposure to extreme weather conditions or inadequate recovery environments, must be assessed to minimize environmental stressors.

By taking a multidimensional approach to risk assessment that incorporates all these elements, we can develop more targeted and effective prevention strategies. This comprehensive approach allows for the identification of all potential risks and ensures that interventions are not only reactive but also proactive, addressing issues before they become significant health problems. Developing good practices in managing the health and well-being of professional soccer players involves engaging all relevant stakeholders, including players, coaches, medical staff, and management, to ensure a collaborative approach.

When developing interventions, it is essential to consider several fundamental principles, such as the ALARA principle (As Low As Reasonably Achievable) and the hierarchy of prevention. These concepts emphasize minimizing risks to the lowest possible level and prioritizing preventive measures, from eliminating hazards to controlling exposure, ensuring that player health and safety are effectively safeguarded.

2. ALARA principle

The ALARA principle, which stands for As Low As Reasonably Achievable, emphasizes that risks should be reduced to the lowest possible level, considering practical limitations such as feasibility, cost, and time. In the context of professional soccer, this means that while it may not be possible to completely eliminate all risks, every effort should be made to minimize them to an acceptable level without compromising player health or performance. For example:

• Load management should be adjusted to prevent overtraining, but in a way that still allows players to maintain peak performance.

• Recovery times should be optimized to minimize the risk of injury while considering the demands of competitive schedules, taking into account not only .

• Mental health resources should be made available to ensure that psychological stress is managed effectively.

3. Hierarchy of prevention

The hierarchy of prevention is a framework used to guide the implementation of safety measures by prioritizing the most effective strategies for reducing risk. It consists of several levels, starting with the most effective form of intervention and working down to less effective methods. The hierarchy typically includes the following steps:

1. *Elimination*: The most effective measure is to completely remove the hazard or risk. In soccer, this could mean restructuring the competition calendar. To achieve this there is a need for a holistic responsibility that transcends the interests of individual clubs, countries and organizers.

2. *Substitution*: If elimination is not possible, the next step is to replace the hazard with something less harmful. For example, replacing high-intensity workouts with lower-intensity or alternative exercises that maintain fitness levels while reducing the risk of overtraining and injury.

3. *Engineering controls*: These involve designing solutions to reduce exposure to hazards, such as I implementing advanced recovery and prevention technologies.

4. *Administrative controls:* This includes changes in policies or procedures to reduce risk, such as I implementing rotation strategies to ensure players get adequate rest or introducing strict recovery protocols. Another example is the design of a global monitoring protocol with clear guidelines for action to safeguard player health.

5. *Personal protective measures*: The least effective but still important measure is equipping individuals with protective resources, such as tailored recovery programs or access to mental health support to mitigate stress.

Such an occupational health-driven approach would enhance overall efficiency by identifying the most impactful measures to protect player health and allowing for continuous evaluation of these interventions. This would create a dynamic, ongoing cycle of assessment, intervention, and re-evaluation, where the effectiveness of each measure is carefully monitored and adjusted as needed. This continuous improvement process is central to occupational health practices and ensures that strategies remain responsive to changing conditions and evolving scientific knowledge.

Conclusion

This study has made it evident that the demands placed on a professional soccer player extend far beyond what happens on the pitch. The physical strain during matches is just one aspect of a much broader picture that includes mental stress, the impact of travel, recovery challenges and many other factors. By thoroughly mapping out these various elements, we can apply occupational health principles more effectively to support players' overall well-being. We can implement strategies that are aimed at improving the general health and well-being of footballers, ensuring that they are not only performing at their best but are also safeguarded against the risks associated with their demanding profession.

Occupational health and safety standards for the football industry: a legal analysis

Prof Frank Hendrickx

Introduction

This analysis takes a legal approach with focus on international and European standards in the field of OSH (Occupational Safety and Health). At the level of intergovernmental organisations, broad and significant legal OSH-frameworks exist. However, they give little to no attention to high-level sports. The International Labour Organisation (ILO) has recently put emphasis on the importance of international labour standards in the world of high performance sport, including OSH standards. The World Health Organisation (WHO) addresses sport through its policy of 'health promotion'. While primarily understood as 'sport for health', as opposed to 'health (and safety) for sport', it stresses the importance of a holistic approach towards health in sport. With reference to major high-level sports events, such as the FIFA World Cup or the Olympics, the WHO recommends a risk-based approach, with risk evaluation, risk mitigation and communication, as well as a consultative decision-making culture.[33]

The main international legal references related to OSH-standards are found in the International Labour Organisation. In a European context, significant standards exist in both the European Social Charter (Council of Europe) as well as EU OSH regulations .

Hereafter, **first**, an outline is given of the relationship between labour law and professional football. The departure point is that professional football players are workers, implying that clubs and professional football players are subject to labour and employment laws. **Second**, an overview of sources and principles is provided that gives account to the fundamental importance of the protection and promotion of occupational health and safety, recognized as a fundamental (human) right, founded in human rights law and included in global and European labour standards, that represent different major legal sources which are applicable to all professional activities and sectors including (professional) football. **Third**, an analysis will be given of major international standards in relation to OSH in professional football, with main references to the ILO and the EU OSH frameworks. This analysis is centered around the areas of alignment with professional football as a sector, in order to understand the practical application of these laws in the context of football.

The objective is to identify the obligations imposed by the holistic approach in the OSH legislation on the relevant stakeholders in the football sector and the implications they bring for these stakeholders to address the risks of football players' workload and risks related to their (mental) health.

---

[33] "Authorities and organizers must undertake adequate planning by taking a risk-based approach to their preparations involving risk evaluation, mitigation and communication. It also requires decision-making on holding and cancelling mass gathering events, and on how to make the event safer by taking a consultative process involving all stakeholders". (https://www.who.int/initiatives/sports-and-health)

Labour standards and professional football

Professional football is a significant industry where football players are, and should be, recognized as workers within a growing global labour market.

The recognition that professional football players are workers is widely shared[34] and essential to understand that rights and obligations resulting from labour laws are upheld in the football sector under both national and international legal frameworks.[35] All major international and European legal sources, as well as the general rule in labour law, indicate that professional football players are workers, who perform work, for remuneration, under the authority of their football club, the employer.[36] This recognition influences various dimensions of professional football, including, among other things, the recognition contracting, wages, working conditions, collective bargaining as well as comprehensive occupational safety and health (OSH) measures that safeguard players' physical and mental well-being.[37]

Labour law scholarship represents 'employment status' of professional sports persons as having a 'dual' character.[38] This means that the employment relationship is governed not only by labour law, but also by sport-specific standards, such as football regulation. This aspect, nevertheless, does not take away that labour law, including its mandatory rules, applies to the football player-club-relationship and thus in the football sector.[39] The foundations of applying labour law, such as addressing vulnerabilities of workers, are also found in the relationship between football players and their clubs/employers.[40]

---

[34] E. WINDHOLZ, "Professional sport, work health and safety law and reluctant regulators", *Sports Law eJournal* 2015, (1) 3; V. SMOKVINA, "New issues in the labour relationships in professional football: social dialogue, implementation of the first autonomous agreement in Croatia and Serbia and the new sports labour law cases", *International Sports Law Journal* 2016, (159) 165; A. DUVAL en O. VAN MAREN, "The Labour Status of Professional Football Players in the European Union: Unity in/and/or diversity?", *ELLJ* 2017, Vol. 8(3), 258 (276); J. KORNBECK, "Bosman and Athlete Welfare: The Sports Law Approach, the Social Policy Approach, and the EU Guidelines on Dual Careers", *Liverpool Law Rev* 2017, (307) 312; B. SCHWAB, "Embedding the human rights of players in world sport", *The International Sports Law Journal* 2018, (214) 217.

[35] M. COLUCCI & F. HENDRICKX, *Regulation employment relationships in professional football*, in European Sports Law and Policy Bulletin, Sports Law and Policy Centre, 2014, 462p.

[36] Some football players working *prima facie* under a self-employed status (in some limited cases) would still be qualified for employment status under international, European or national law: cf. A. DUVAL & O. VAN MAREN, "The Labour Status of Professional Football Players in the European Union: Unity in/and/or diversity?", *ELLJ* 2017, Vol. 8(3), 260-267, and: "In the case of professional football players, a wealth of legal and factual indicia weigh in favour of identifying them as employees under the ILO and the CJEU's definitions of the concept" (*Ibidem*, p. 277).

[37] E. WINDHOLZ, "Professional sport, work health and safety law and reluctant regulators", *Sports Law eJournal* 2015, (1) 3.

[38] C. PAPADIMITRIOU, "The particularities of the 'job' performance of the professional athlete: rights and obligations of the parties to the employment contract", in J. PICHRT & K. KOLDINSKA (eds.), *Labour law and social protection in a globalised world*, *Bulletin of Comparative Labour Relations*, Vol. 103, Kluwer Law International, Alphen aan den Rijn, 2018, 168.

[39] C. PAPADIMITRIOU, "The particularities of the 'job' performance of the professional athlete: rights and obligations of the parties to the employment contract", in J. PICHRT & K. KOLDINSKA (eds.), *Labour law and social protection in a globalised world*, *Bulletin of Comparative Labour Relations*, Vol. 103, Kluwer Law International, Alphen aan den Rijn, 2018, 167-168; S. BELLOMO, "Conclusion and termination of employment relationship in professional sports: special regulations and paticular protection requirements", in J. PICHRT & K. KOLDINSKA (eds.), *Labour law and social protection in a globalised world*, *Bulletin of Comparative Labour Relations*, Vol. 103, Kluwer Law International, Alphen aan den Rijn, 2018, 160.

[40] V. SMOKVINA, "New issues in the labour relationships in professional football: social dialogue, implementation of the first autonomous agreement in Croatia and Serbia and the new sports labour law cases", *International Sports Law Journal* 2016, (159) 165.

Like workers in any other sector entering into an employment contract, labour laws are primarily mandatory laws and arising from fundamental rights protection. They outline and impose rights and obligations within the contractual relationship between players and clubs. Despite the unique nature of their work, high performance football requiring intense physical and mental demands and pressure, players are, as a general rule, entitled to the same overall legal protections as workers in other industries. While football has its own governing rules, such as those set by football governing bodies (including FA's, FIFA and UEFA), these do not override national labour laws or the rights that players have under these laws.

The application of labour law in sport does not necessarily mean that the specificity of sport cannot be recognised. The European Union (EU) has recognized the specificity of sport, particularly through the *Nice Declaration* of 2000, which states sport's social, educational, and cultural functions must be considered.[41] It is also reflected in article 165 of the TFEU. The specificity of sport is nevertheless to be guaranteed within (and not outside) the boundaries of the law. It would, however, allow the application of "a case-by-case inspection of sport practices (…) with receptivity to the particular features of sport that may justify practices that would not be tolerated elsewhere", rather than exempting sport from applicable legal rules.[42] For example, there will be acceptance, also in the context of OSH standards, of high performance standards and the organisation of work in an exceptional high-level sport environment, in light of the specific characteristics of football. At the same time, "specifity should not mean that sport operates outside the law, nor that the hierarchy of legal norms can be disregarded".[43] While the need for adaptation and tailoring of OSH standards to the specific sport context is recognised, it cannot undermine the fundamental protection or rights of football players as workers, neither can they set aside mandatory working conditions imposed by public regulation under labour and employment laws.[44]

The application of labour law to professional football is, therefore, also about ensuring that the working conditions of players meet the applicable labour standards. Occupational Safety and Health (OSH) is a critical component of labour law that directly impacts the well-being of workers[45] and it considered as a fundamental right.

---

[41] R.C.R. SIEKMANN, *Introduction to International and European Sports Law. Capita Selecta*, The Hague, Asser Press, 2012, 72.

[42] S. WEATHERILL, "The Lex Sportiva and EU law: the acaemic lawyer's path before and after *Bosman*", in A. DUVAL & B. VAN ROMPUY (eds.), *The legacy of Bosman. Revisting the relationship between EU law and sport*, Asser International Sports Law Servies, Springer & TMC Asser Press, The Hague, 2016, 243.

[43] F. HENDRICKX, "When sport and labour law are interlocked: playing ball with modernisation", in J. PICHRT & K. KOLDINSKA (eds.), *Labour law and social protection in a globalised world, Bulletin of Comparative Labour Relations*, Vol. 103, 155.

[44] S. BELLOMO, "Conclusion and termination of employment relationship in professional sports: special regulations and paticular protection requirements", in J. PICHRT & K. KOLDINSKA (eds.), *Labour law and social protection in a globalised world, Bulletin of Comparative Labour Relations*, Vol. 103, 163.

[45] B.O. ALLI, Fundamental Principles of Occupational Health and Safety, Geneva, International Labour Office, 2008, vii.

OSH as a fundamental human right

1. UN and ILO's framework

Occupation health and safety (OSH) is a guarantee based in international human rights law. The United Nation's International Covenant on Economic, Social and Cultural Rights of 1966 (ICESCR), puts human dignity central to labour standards at work.[46] This foundation is also confirmed in the International labour organisation (ILO)'s founding principle that 'labour is not a commodity'.[47]

The ICESCR requires states to recognize 'the right of everyone to the enjoyment of just and favourable conditions of work which ensure, in particular, safe and healthy working conditions (article 7, b). It also guarantees the right to "rest, leisure and reasonable limitation of working hours and periodic holidays with pay, as well as remuneration for public holidays" (article 7, d). It furthermore guarantees the right of everyone to the enjoyment of the highest attainable standard of physical and mental health (article 12, 1).

The state-parties agreed (in article 23) that the international human rights of the ICESCR require further international action for the achievement of these rights, including the conclusion of conventions and the adoption of recommendations. In light of this, the International Labour Organisation (ILO) has adopted further and more detailed OSH standards.

The ILO's framework confirms the fundamental status of occupation health and safety as a human right. In 1998, the general conference of the ILO (the ILC) adopted the Declaration on Fundamental Principles and Rights at Work. All ILO Member States undertake to respect, to promote and to realize, in good faith, the principles and rights concerning a few core fundamental labour standards: freedom of association and collective bargaining, the elimination of forced labour, the abolition of child labour, and the elimination of discrimination in employment and occupation. In 2022, the ILO incluced in this list of core fundamental principles, the guarantee of a safe and healthy working environment. The ILO Conventions n° 155[48] and n° 187[49] have, as a consequence of this, been identified as part of the most fundamental instruments of the ILO. Since the ILO is a tripartite organisation, with a representation of employers' and union organisations in the International Labour Conference (ILC), it implies that it concerns a broad stakeholder commitment to these fundamental standards.

2. European human rights standards

In a European context, the European Social Charter (1961, revised in 1996) provides a key instrument in the European human rights system. It lays down the fundamental guarantee for health and safety at work for all workers. With the European Union evolving, OSH has also become a more central feature of the social dimension of the EU and one of the most important realisations in the regulations of EU social policy. Also within the EU legal order, OSH is recognised as a fundamental right. This is encapsulated in the Charter of Fundamental Rights of the European Union (CFREU), particularly in Articles 31, 32, and 35, and is also reflected in Principle 10 of the European Pillar of Social Rights (EPSR).

---

[46] Preamble of the International Covenant on Economic, Social and Cultural Rights (ICESR).
[47] Article 1(a) ILO Declaration of Philadelphia, Geneva, 1944.
[48] Occupation Safety and Health Convention n° 155, Adoption: Geneva, 67th ILC session (22 Jun 1981).
[49] Promotional Framework for Occupational Safety and Health Convention n° 187, Adoption: Geneva, 95th ILC session (15 Jun 2006).

International and European OSH standards

1. International Labour Standards (ILO)

The International Labour Organisation (ILO), a specialised UN agency, is the main standard setting organisation at international level for OSH standards. Standards receive the form of conventions (binding treaties under international law) and recommendations.

The ILO has not yet issued specific guidance for the world of sports. However, it has launched a sport policy programme in which it pays attention to the importance of looking to (professional) sport as work and the relevance of the five core fundamental rights of the ILO, including health and safety protection.

In summary, the ILO identifies a number of risks for professional sport:[50]

- Athletes, a diverse group, face significant risks of injury and long-term physical and mental health issues, often greater than those in many other professions. The sports sector generally accepts a higher threshold for occupational risks, focusing on risk management rather than elimination. Athletes, driven by external pressures from coaches and management, may prioritize performance over safety, sometimes concealing injuries to continue competing.
- Elite athletes, in particular, face increasing risks of enduring injuries due to intense competition schedules. These demands, coupled with insufficient recovery time, can shorten careers and negatively impact both physical and mental health.
- Mental health risks are prevalent among athletes, exacerbated by physical demands, competition stress and cultural factors. Many athletes avoid seeking mental health support due to busy schedules, past negative experiences, and cultural barriers.
- Doping remains a serious concern, endangering both the integrity of sports and athletes' health.
- Additionally, athletes are at increased risk of interpersonal violence, including psychological, physical, and sexual abuse, often stemming from coaches, authority figures, or peers.

In response to these risks, the ILO refers to its key OSH labour standards, including Convention n° 155 (OSH Convention), Convention n° 161 (Occupational Health Services Convention) and Convention n° 187 (OSH Promotional Framework Convention) as well as related recommendations, including the Occupational Safety and Health Recommendation n° 164.

Convention 155 introduced a renewed approach to OSH, implying that health and safety is more than the absence of accidents or illness, requires attention to both physical and mental aspects of work[51] and implies a global and multidisciplinary approach in terms of occupational health and safety risk assessment.[52] Convention 187 has a focus on promoting occupational health and safety, to see it as a priority with the development and fostering of a 'culture' of prevention.

---

[50] ILO Technical Brief, *Professional athletes and the fundamental principles andrights at work*, May 2024, ILO, Geneva, 22-24.
[51] Article 3 C155 - Occupational Safety and Health Convention (ILO Convention No. 155), 1981.
[52] Article 6 C155 - Occupational Safety and Health Convention (ILO Convention No. 155), 1981.

Several more specific ILO conventions and recommendations deal with specific branches of activity[53] and others concern a number of specific categories of workers. However, sport has not been included in these approaches. This does not mean that sport would be excluded. The scope of application of the ILO norms is broad and includes all those who are in work, regardless of the activity. According to article 1 of Convention 155, the standards apply to all branches of economic activity and, according to article 2 of this convention, to all workers. The OSH standards of the ILO are, therefore, also applicable to the professional football sector.

The broad field of applications of ILO's OSH standards goes along with flexibility of the standards. This gives room to ensure that ILO standards are tailored to the specific needs and circumstances of specific sector, such as sport or professional football. The supervising committees of the ILO have confirmed that specificities of work or context cannot be a ground to deny the OSH-standards.[54] The main message is that OSH standards should be applied and taylored to the specific characteristics and context of the work activity.

2. European Social Charter

In a European context, a main relevant instrument comes from the Council of Europe. The European Social Charter (ESC 1961, revised in 1996) delivers an important guarantee of OSH standards, providing for a fundamental right to safe and healthy working conditions.[55]

Within the interpretation of the ESC there is a trend towards strengthening all-round prevention taking into account physical and mental well-being of workers".[56] The ESC's OSH standards apply to all workers, all workplaces and all sectors of activity.[57]

The supervising committee of the ESC considers the implementation of both the EU's as well as the ILO's OSH standards as important indicators to determine whether or not the European standards are respected.[58]

3. EU OSH legislation

The primary legal foundation for occupational safety and health (OSH) regulation within the European Union is the OSH Framework Directive (OSHFD). This directive is designed to enhance worker safety and health across all workplaces by prioritizing the human aspects of occupational

---

[53] The following instruments included in the annex to R197 - Promotional Framework for Occupational Safety and Health Recommendation (ILO Recommendation No. 197), 2006: C120 - Hygiene (Commerce and Offices) Convention (ILO Convention No. 120), 1964 and R120 - Hygiene (Commerce and Offices) Recommendation (ILO Recommendation No. 120), 1964; C167 - Safety and Health in Construction Convention (ILO Convention No. 167), 1988 and R175 - Safety and Health in Construction Recommendation (ILO Recommendation No. 175), 1988; C176 - Safety and Health in Mines Convention (ILO Convention No. 176), 1995 and R183 - Safety and Health in Mines Recommendation (ILO Recommendation No. 183), 1995; C184 - Safety and Health in Agriculture Convention (ILO Convention No. 184), 2001 and R192 - Safety and Health in Agriculture Recommendation (ILO Recommendation No. 192), 2001; C152 - Occupational Safety and Health (Dock Work) Convention (ILO Convention No. 152), 1979 and R160 - Occupational Safety and Health (Dock Work) Recommendation (ILO Recommendation No. 160), 1979.

[5454] J.-M. SERVAIS, *International labour law*, 8th edition, in *International Encyclopaedia of Labour Law and Industrial Relations* (ed. Frank Hendrickx), Wolters Kluwer, Alphen aan den Rijn, 687-694.

[55] Article 3 European Social Charter (ESC).

[56] R. BIRK, *European Social Charter*, in International Encyclopaedia of Labour Law and Industrial Relations (ed. Frank Hendrickx), Wolters Kluwer, Alphen aan den Rijn, 2007, p.110 (nr.95).

[57] Digest of the case law of the European Committee of Social Rights, Council of Europe, Strasbourg, 2022, 64. (www.coe.int), 68.

[58] Digest of the case law of the European Committee of Social Rights, Council of Europe, Strasbourg, 2022, (www.coe.int), 64.

health. The OSHFD establishes broad, goal-oriented standards applicable to all sectors and types of workplace risks, with general minimum requirements.

A notable characteristic of the OSHFD is its reliance on general, adaptable provisions. This approach allows for flexibility in interpretation, accommodating changing circumstances and tailoring to specific needs and contexts. The focus of the legislation is on setting objectives, leaving the responsible actors to determine the best methods for achieving compliance.

Beyond the OSHFD, several individual EU directives offer more detailed regulations, targeting specific risks, sectors, or vulnerable worker groups. These individual directives build on the general principles set out by the OSHFD, providing specific, sometimes technical, requirements. There is, however, no reference to sport or football in these more specific standards.

Tailoring OSH to professional football

The OSH standards in the various legal instruments, mentioned above, contain a variety of obligations and principles. The broad set of legal obligations and principles, although sometimes rather precisely formulated, have an abstract character and possess a high degree of flexibility. The purpose of this is to recognise that OSH standards need to be tailored. The scope of OSH standards is wide, covering all workers and all sectors of activities, but they need to be implemented taking into account the specific circumstances and characteristics of the work and the context in which it is performed. It also means that OSH standards need to be tailored to the specific context of professional football.

In order to illustrate this, a few thematic OSH issues are provided hereafter. For each of them, the EU-OSHFD standards are taken as departure point, in order to show that their sometimes detailed character still have room, but also potential, for a professional football-oriënted translation and implementation.

1. Scope of application

Also within the EU's OSHFD, all workers in all sectors of activity are covered by the provisions and the obligations arising from them.[59] All standards thus apply to workers in the sport sector, including professional football players.

The OSHFD leaves not much room for exceptions. There is a possibility to deviate from the standards for "public service" activities. This relates activities which, because of their peculiar characteristics, conflict with the provided OSH standards. It does not concern an open justification for deviation. The directive itself mentions the armed forces, the police, or civil protection services, as activities of 'public service'. Any exception that would justify the non-application of OSH standards would need to rely on this public service argument, whereby it is required that the services provided emanated from 'public authority'. The European Court of Justice has confirmed this in the *Sindicatul Familia Constanța*-case. Some exceptions could be granted to the employment of foster parents, who are considered as workers and who are in principle available 24/7 to take care of foster children in their own households. They can, however, only be excluded from the scope of the EU OSHFD, if they are seen as performing a "public sevice". The Court also held that,

[59] Article 2 Council Directive of 12 June 1989 on the introduction of measures to encourage improvements in the safety and health of workers at work (89/391/EEC) - OSH "Framework Directive" (OSHFD).

even if an exception is recongised, it is still required to ensure that the safety and health of workers is guaranteed "as far as possible" in light of the objectives of the European OSHFD.[60]

The scope of the OSHFD is similarly broad in relation to the specific risks that are concerned in relation to work. There is a duty to ensure the safety and health of workers in every aspects related to the work.[61] Furthermore, the CJEU has consistently held that the OSHFD applies to all cases, regardless of the size of the employer or of the number of their workers.[62]

The OSHFD recognises that there may be groups of workers with specific risks or vulnerabilities, which have to be taken into account in the development and implementation of OSH policies. Particularly sensitive risk groups must be protected against dangers which specifically affect them.[63] One may think about young workers, pregnant workers, breastfeeding workers, or more broadly, the gender-dimension or ageing dimension of OSH.

Implications for professional football:

- Professional football players are considered workers under international and European OSH standards.
- This means that clubs and other relevant stakeholders must adhere to OSH regulations, ensuring that health and safety are managed, as it is for other workers in any other industry.
- Pay attention to specific vulnerable groups of players (e.g. young players, gender dimension).

2. OSH responsibility

Under the OSH standards, the employer has the main duties to ensure the safety and health of workers in every aspect related to the work.[64] While workers may have, to some degree, an own responsibility in the field of safety and health at work, this does not affect the principle of the responsibility of the employer.[65]

The employer's obligations to ensure workers' health and safety are extensive, requireing all necessary precautions and measures be taken. OSH standards require a dynamic approach to occupational safety and health, requiring employers to consistently update and refine their safety measures in response to evolving circumstances.[66]

Like many other fields of sports, professional football is regulated by many different sport governing bodies, such as national football associations, professional football leagues and international regulators, such as UEFA or FIFA. This could mean different things in relation to OSH standards. Football regulations intersect with working conditions and would thus need to be aligned with OSH standards. Football governing bodies may be required to contribute to a (shared) responsibility for OSH standards in professional football. The Council of Europe's European Sports

[60] CJEU 20 November 2018, nr. C-147/174, ECLI:EU:C:2018:926, Sindicatul Familia Constanţa, §78.
[61] Article 5(1) OSHFD.
[62] CJEU 7 February 2002, nr. C-5/00, Commission of the European Communities/Germany.
[63] Article 15 OSHFD.
[64] Article 5(1) OSHFD.
[65] Article 5(3) OSHFD.
[66] Article 6 OSHFD.

Charter (Revised versions 2021)[67] emphasises the role and responsibility of the whole sport sector, and in particular sport governing bodies, to implement internationally recognised fundamental human rights standards.[68] Furthermore, the level of football governing bodies may be a more appropriate level to organise expertise, consultation, dialogue and regulation about OSH standards, tailored to the specific needs, circumstances and characteristics of football and the professional football sector. Recent case law of the CJEU shows that football governing bodies, such as those with international regulatory power, like FIFA and UEFA, have the power to regulate, but also control, the participation of players and clubs in sporting competitions.[69]

In light of this, specific mention has to be made of social dialogue, discussions, negotiations and agreements, between workers' representatives (players' unions) and employers (clubs), to establish and implement OSH standards and policies at sector-level. In the framework of European social dialogue (art. 155 TFEU), many examples exist of sector-level tailoring and specification of international and European OSH standards,[70] amongst which sector-level agreements.[71]

Implications for professional football:

- Clubs need a comprehensive OSH policy covering all aspects of player and staff safety.
- Sufficient resources need to be available to implement and maintain OSH policies effectively (this includes dedicated personnel and equipment to meet the safety needs of players and staff).
- Football governing bodies may assume a shared responsibility in establishing an overall OSH-culture and to support the development and implementation of OSH standards and make sure they are tailored to the specific needs and characteristics of the football sector.
- Social dialogue is a suitable way to set and implement specific and tailored standards for the football sector

3. Prevention and risk assessment

Prevention and risk assessment are a key focus of OSH standard. This is recognized as the foundational principles of OSH measures. Prevention is broadly defined as actions aimed at preventing or mitigating occupational risks.[72]

Article 6(2) OSHFD further elaborates on the various components of this overarching prevention principle, offering a detailed framework for employers to follow in their OSH strategies. These

---

[67] Council of Europe,'Revised European Sports Charter', Recommendation CM/Rec(2021)5 Adopted by the Committee of Ministers on 13 October 2021 at the 1414th meeting of the Ministers' Deputies, https://rm.coe.int/revised-european-sports-charter-web-a6/1680a7534b

[68] Cf. Articles 2 (scope), 4 (governance) and 6 (human rights) of the Revised European Sports Charter.

[69] C-333/21, European Super League Company v. FIFA & UEFA (Grand Chamber), CJEU 21 December 2023, cf. para. 142.

[70] Cf. the EU social dialogue texts database: https://ec.europa.eu/social/main.jsp?catId=521&langId=en&day=&month=&year=&sectorCode=&themeCode=DOMS08&typeCode=&recipientCode=&mode=searchSubmit&search=Search

[71] Examples of specific binding EU sector-level instruments: exposure to silica at work for construction and extraction workers (Agreement on Workers Health Protection through the Good Handling and Use of Crystalline Silica and Products containing it, *OJ C 279, 17.11.2006) and* exposure to specific dangers and injuries in the hospital and healthcare sector (Council Directive 2010/32/EU of 10 May 2010 implementing the Framework Agreement on prevention from sharp injuries in the hospital and healthcare sector concluded by HOSPEEM and EPSU (Text with EEA relevance), *OJ L 134, 1.6.2010).*

[72] Article 3(d) OSHFD.

include: avoidance of risks,[73] evaluation risks which cannot be avoided,[74] combating the risks at source,[75] adapting the work to the individual,[76] adapting to technical progress,[77] giving collective protective measures priority over individual protective measures.[78] These specific obligations can have various meanings for professional football and a reflection will need to be undertaken about the meaning of these specific obligations and their tailoring to the sector.

Implications for Professional Football:

- prevention strategy and regular risk assessment: associated with all kinds of work related risks, such as training and playing conditions, facilities, equipment, reduction of injury risks, and implement measures to mitigate these risks, taking into account a high performance context.
- assess risks that may be inherent to the sport of football, such as contact injuries, the impact of intensive training periods and matches, and implement strategies to manage these risks.
- minimize risks by optimizing training methods, field and game conditions, implement protocols to avoid risks such as injuries, avoid excessive workload, prevent player fatigue and ensure recovery periods, taking into account individual circumstances of players.
- address risk factors directly at their source, such as improving design and planning, including equipment to reduce injury risks, adapt training techniques to prevent overuse injuries, adapt formats and planning in order to adapt work-overload.
- prioritize team-wide safety measures, such as improving overall training and match scheduling policies, over individual protective equipment.
- utilize the latest advancements in sports science and technology to enhance player safety, such as innovations in injury prevention equipment, data analytics for managing player effort and workload in light of an OSH strategy.
- use the potential of sector regulation and standardisation which could be delivered from football governance organisations.

4. Worker involvement

Worker involvement is an essential component of occupational safety and health (OSH), particularly in high-stakes, fast-paced environmenst such as professional football. By involving players and their reprsentative in decisions that directly impact their safety and well-being, clubs and associations can create more effective and relevant health and safety policies. This approach is supported by the International Labour Organization (ILO) and specifically outlined in ILO Convention No. 155, article 20, which underscores the importance of giving workers and their representatives a voice in OSH matters.

Worker involvement requires a meaningful role in shaping, implementing, and overseeing health and safety policies. This involvement extends to decisions about critical aspects such as training schedules, injury prevention protocols, and match planning and timing, all of which have significant implications for players' health.

[73] Article 6, Paragraph 2(a) OSHFD.
[74] Article 6, Paragraph 2(b) OSHFD.
[75] Article 6, Paragraph 2(c) OSHFD.
[76] Article 6, Paragraph 2(d) OSHFD.
[77] Article 6, Paragraph 2(e) OSHFD.
[78] Article 6, Paragraph 2(h) OSHFD.

The importance of this participation lies in ensuring that health and safety measures are not merely imposed top-down, but are developed with direct input from those most affected by them.

As mentioned above, social dialogue is a suitable way to involve workers' representatives (players' unions) and employers' organisations (clubs representatives) in the establishment of sector-specific OSH standards and policies.

Implications for Professional Football

- Players and their representatives should be actively involved in the development of OSH policies at the relevant football governing levels where decisions or regulations about or impacting OSH are developed.

5. Occupational health services

The OSH standards address the need for health surveillance to protect workers from health and safety risks associated with their work.[79] Those standards have been designed for all kinds of activity and are also applicable to the sport sector. However, it is important to take into account the specific context of high performance sport. In a high performance environment, such as professional football, health monitoring needs to be organised in relation to the increased risk environment.

Implications for Professional Football:

- Health surveillance: ensure that players are monitored and have access to health surveillance, especially given the high performance context and the associated risks.
- Player oriented health monitoring: apply health monitoring in light of OSH strategies, in relation to risks that could affect a player's overall well-being, and not only in relation to criteria of sporting (high) performance.

Conclusion

This legal analysis has demonstrated that Occupational Safety and Health (OSH) standards, as defined by international frameworks, are fully applicable to the professional football industry. Professional football players are recognised as workers under both international, European and national labour law, and are entitled to the same guarantees as other workers. This legal recognition places responsibilities on the whole football sector, in particular football clubs (the employers) and governing bodies (the organizers), to ensure that players' safety and health are addressed and managed effectively.

Central is the legal recognition of OSH as a fundamental human right, a principle affirmed by international instruments such as the United Nations, the International Labour Organization (ILO), the Council of Europe and the European Union. This recognition covers both the physical and mental health of players, underscoring the need for comprehensive regular risk assessments and the development of preventive measures.

The OSH regulatory framework can and should be tailored to the specific demands and characteristics of professional football. The flexibility of OSH standards allow for adaptation to the

[79] Cf. Article 14 OSHFD.

unique characteristics of professional sports, particularly the high performance context and physical demands, including the risks associated with it.

While the need for adaptation and tailoring of OSH standards to the specific sport context is recognized, however, it cannot undermine the fundamental protection or rights of football players as workers, neither can they set aside mandatory working conditions imposed by public regulation under labour and employment laws. The supervising committees of the ILO have confirmed that specificities of work or context cannot be a ground to deny the OSH-standards.

For the football sector, compliance with OSH standards necessitates a proactive and holistic approach to player welfare. In this context, worker involvement in the creation and implementation of OSH policies is essential and built-in as a legal requirement of the OSH standard frameworks.

Key Findings

1. **Compliance with Legal Framework and OSH Standards**
    - Professional soccer players are legally acknowledged as employees, thus subject to labour laws and OSH regulations that govern worker rights.
    - Soccer clubs, federations, and other stakeholders have an obligation to adhere to established OSH frameworks, including the ILO conventions, the European Social Charter, and EU OSH standards.
    - These laws mandate a structured approach to safeguard player health, ensuring that clubs take concrete steps to protect athletes under universally recognized labour protections.
2. **Holistic Occupational Health and Safety Standards in Football industry**
    - OSH regulations in soccer address both the physical and mental well-being of players, acknowledging the intense demands unique to professional sports.
    - While adjustments in health and safety standards may accommodate the sport's performance demands, fundamental worker protections remain non-negotiable to safeguard athlete rights.
    - This approach mandates that all standards encompass the complete spectrum of player health needs, which is essential to their resilience and long-term career viability.
3. **Comprehensive Risk Management Strategy**
    - Effective OSH implementation in soccer necessitates multifaceted risk assessments that go beyond match or training loads.
    - Assessments should consider four key dimensions:
        1. **Job Content**: Physical exertion, mental resilience, and psychological demands;
        2. **Work Relations**: Quality of team dynamics and support systems;
        3. **Work Environment**: Factors like extensive travel schedules and on-field conditions;
        4. **Organizational Aspects**: Job security, contract clarity, and career stability.
    - Using the Job Demands-Resources (JD-R) model, clubs can evaluate these areas holistically and tailor interventions to mitigate risks, ensuring long-term player health and optimal performance.
4. **Benefits of Integrating OSH for Player Welfare**

   - Embedding OSH within soccer institutions offers a structured approach to safeguarding players’ health, reducing injury risks, and supporting extended, sustainable careers.
   - By continuously assessing and improving health and safety practices, clubs foster an environment of growth, resilience, and well-being, directly benefiting players and the sport as a whole.
   - This not only complies with legal obligations but also optimizes players’ professional longevity and productivity through proactive health management.

5. **Necessity for Collective Action and Organizational Accountability**
   - While individual clubs often initiate OSH practices, comprehensive protection requires a sector-wide commitment involving leagues, federations, and governing bodies. Collaboration among clubs, leagues, and governing bodies is essential for systemic solutions to health risks, thereby building a framework that prioritizes collective responsibility for the holistic well-being of soccer players. As employers, clubs hold primary responsibility for health and safety; however, the industry’s structural aspects (e.g., competition calendars, contractual arrangements) also impact player welfare.
   - Worker involvement is an essential component of occupational safety and health (OSH), particularly in high-stakes, fast-paced environments such as professional football. It requires a meaningful role in shaping, implementing, and overseeing health and safety policies. This involvement extends to decisions about critical aspects such as prevention protocols, match planning and timing, all of which have significant implications for players' health.
   - Collective agreements with social partners, facilitated through established mechanisms like social dialogue at the European or International level, help achieve sectoral agreements and standardization within a transnational industry.

These messages underline that OSH regulations in professional soccer are essential not only as legal imperatives but also as strategic priorities. A coordinated, holistic approach ensures compliance, promotes health, and sustains the athletic workforce in a way that supports both player welfare and professional success.
