# Supplementary material for: The International Match Calendar in Men's Professional Football: An Expert Position Statement
Source: Scand J Med Sci Sports. 2025 Nov 6;35(11):e70163. doi: 10.1111/sms.70163 (PMC12591028; doi:10.1111/sms.70163)
Supplement: Supplementary file 3 — Data S3: sms70163‐sup‐0003‐Supinfo3.docx. [file SMS-35-e70163-s002.docx]

Supplemental file C

Table SC1. Fixture congestion studies

| Study | Sample | Research Design | IMC Indicator | Outcome Measure | Key Findings |
| --- | --- | --- | --- | --- | --- |
| Bengtsson, Ekstrand et al. (2013) | 8150 Games from 27 Teams playing in UEFA competitions and 10 European domestic leagues | Retrospective cohort (11-year follow-up) | Acute and Chronic Fixture Congestion | Injury and Performance | Total and muscle injury rates increased in matches with short recovery periods (<5 days) compared to matches with longer recovery times (>5 days).  High match load was associated with increased muscle injury rates in the same period and increased ligament injury rates in subsequent periods.  Match congestion had no significant impact on overall team performance​. |
| Bengtsson, Ekstrand et al. (2018) | 133170 Match Observations in UEFA competitions and 16 European domestic leagues | Prospective cohort (14-years) | Acute and Chronic Fixture Congestion | Injury | Muscle injury rates were 21% lower when players had 6 days between matches compared to ≤3 days.  No significant differences were found in overall injury rates based on short-term or long-term match congestion. |
| den Hollander, Kerkhoffs et al. (2024) | 860 Footballers playing in top 5 European Leagues, UEFA competitions and/or international fixtures (mean age 26) | Retrospective case-control over two seasons (2021-2023) | Acute and Chronic Fixture Congestion and Travel | Injury | Injured players had higher match workloads and travelled further across more time zones compared to non-injured players. |
| Lago-Penas (2009) | 180 La Liga & Champions League games | Retrospective cohort | Acute Fixture Congestion | Performance | Playing a midweek UEFA Champions League match did not significantly affect team performance in the domestic league. Contrary to expectations, UEFA Champions League debutants performed above their normal standard in the weekend matches following a midweek match. |
| Lago-Penas, Rey et al. (2011) | 172 La Liga Footballers | Retrospective case-control | Acute Fixture Congestion | Performance | Players who played two matches per week covered less distance at higher intensities (maximal, submaximal, medium) compared to those who played one match per week, although differences were not significant. |
| Rey, Lago-Ballesteros et al. (2010) | 42 La Liga Players | Retrospective cohort | Acute Fixture Congestion | Performance | No significant differences were found in the total distance covered or in high-intensity activity across successive matches during a congested fixture period. |
| Silva (2021) | 19 Premier League Players (mean age 27) | Retrospective cohort study | Acute Fixture Congestion | Performance | The number of matches in a week did not significantly affect total distance or high metabolic load in significant ways. |
| Dellal, Lago-Peñas et al. (2015) | 16 Footballers playing in Ligue 1 and UEFA champions League (mean age 24) | Retrospective cohort | Acute Fixture Congestion | Injury and Performance | Physical and technical performances did not differ across games during congested periods, but match injury rates increased significantly​. |
| Dupont, Nedelec et al. (2010) | 32 Elite French, Scottish and Norwegian footballers (mean age 26) | Retrospective cohort | Acute Fixture Congestion | Injury and Performance | Playing 2 matches per week does not significantly affect match-related physical performance (total distance, high-intensity distance, sprint distance, and number of sprints). However, the injury rate was significantly higher for players who played 2 matches per week compared to those who played 1 match per week (25.6 injuries per 1000 hours vs. 4.1 injuries per 1000 hours). |
| Carling, Gall et al. (2010) | 31 Footballers competing in Ligue 1 and UEFA competitions | Prospective cohort | Acute Fixture Congestion | Injury | Players in congested fixtures (≤3 days between games) did not experience higher injury rates. |
| Carling, McCall et al. (2016) | 25 UEFA Champions League and Europa League Footballers | Prospective cohort | Acute Fixture Congestion | Injury | Greater injury risk, especially during the final 15 minutes of matches in congested periods.  Higher likelihood of injuries such as ankle sprains and non-contact injuries in congested match cycles.  Muscle strains were frequent, particularly hamstring injuries, during the latter stages of congested matches. |
| Howle, Waterson et al. (2020) | 42 Players from Australian A League (mean age 26) | Prospective cohort | Acute Fixture Congestion | Injury | Injury rates were significantly higher during multi-match weeks compared to single-match weeks, with training and match injury risks increasing during congested schedules.  Internal and external markers of load were also significantly reduced during congested weeks and seasons, though despite these reductions, the risk of total injury and significantly training based injuries was still increased during fixture congestion. |
| Clemente, Mendes et al. (2017) | 35 Players from Portugues Premier League (mean age 26) | Repeated measures cohort | Acute Fixture Congestion | Health/Wellness | Weeks with two official matches showed significantly higher levels of fatigue, muscle soreness (DOMS), and overall internal training load scores compared to weeks with one match. |
| Morgans, Orme et al. (2014) | 21 Premier League players (mean age 26) | Repeated measures cohort | Acute Fixture Congestion | Health/Wellness | Significant reduction in salivary IgA after congested games, but levels returned to normal when regular game intervals resumed.  Suggests mucosal immunity is affected by congested fixture schedules, which may increase illness risk. |
| Howle, Waterson et al. (2019) | 42 Footballers playing in Australian A-League and AFC Champions League (mean age 26) | Prospective cohort | Acute Fixture Congestion | Health/Wellness | Players experienced reduced recovery profiles in multi-match weeks compared to single-match weeks, with significant reductions in wellness scores and physical performance following congested schedules. |
| Lundberg and Weckstrom (2017) | 16 Veikausliiga (Finland) footballers (mean age 25) | Retrospective case-control | Acute Fixture Congestion | Health/Wellness | Muscle soreness was 42% higher in congested players compared to controls.  Some biochemical markers (e.g., P-Urea) were elevated in congested players, indicating physiological stress.  However, explosive physical fitness (e.g., sprint, jump performance) was not compromised by fixture congestion. |
| Delaval, Abaidia et al. (2022) | 46 Ligue 1 players (mean age 24) | Retrospective cohort over two seasons (2015-2017) | Acute Fixture Congestion and Rest/Recovery | Injury | During a congested schedule, noncontact injuries were associated with fatigue and muscle soreness, especially 2-3 days after matches. Recovery protocols involving sleep, fatigue, and muscle soreness monitoring, and isometric force tests helped reduce injury risk during congested schedules​. |
| Noor, McCall et al. (2021) | 37 Footballers playing for the Australian National team (mean age 26) | Retrospective cohort | Acute Fixture Congestion and Rest/Recovery | Health/Wellness | During international tournaments, perceived load and recovery profiles were largely influenced by exposure to match play.  Acute match congestion (2 matches within 4 days) impaired players' pre-match perceived status compared to non-congested microcycles but did not exacerbate post-match fatigue and recovery responses |
| Pinheiro, Quintao et al. (2023) | 13 Footballers playing in Brazilian First Division (mean age 29) | Prospective cohort | Chronic Match Congestion | Injury, Performance | Muscle injury incidence was higher during the congested match period. Despite the increased injury risk, no significant differences in physical performance or fatigue metrics were observed between matches separated by 3–4 or 6–7 days |
| Carling, Le Gall et al. (2012) | 19 Footballers playing in Ligue 1 and Europa League | Prospective cohort | Chronic Match Congestion | Injury and Performance | Overall and light-intensity distance run varied, but high-intensity running performance remained stable across games.  Injury rates during the congested period were similar to outside periods, but the duration of injuries was shorter. |
| Ekstrand, Waldén et al. (2004) | 266 Footballers playing in top UEFA leagues and in the World Cup (mean age 26) | Prospective cohort | Chronic Match Congestion | Injury and Performance | World Cup players played significantly more matches during the season compared to non-World Cup players (46 vs. 33 matches).  There was no increased risk of injury for World Cup players during the season.  29% of World Cup players incurred injuries during the tournament, and 32% underperformed.  Players who underperformed had played more matches in the 10 weeks leading up to the World Cup compared to those who performed better (12.5 vs. 9 matches).  A congested match calendar can lead to fatigue, increasing the risk of injury and underperformance in subsequent periods. |
| Dalen-Lorentsen, Andersen et al. (2022) | 421 player seasons from Norwegian male football premier league | Prospective cohort over 2 consecutive seasons | Chronic Match Congestion | Injury | Both seasons had 30 game weeks, but one was 57 days shorter (due to COVID). No significant difference in match injury rate between seasons. No differences in the number of injuries, days lost to injury, matches missed to injury, or injury severity between the two seasons. |
| Lackner and Sonnabend (2023) | 13537 match observations from Bundesliga, La Liga and Serie A | Retrospective cohort using Cox proportional hazard model | Chronic Match Congestion | Injury | Players in high-workload teams returned from injury earlier than expected, but this increased the risk of subsequent injuries. High workload and pressure from team performance contributed to returning earlier from injury. |
| Moreno-Perez, Paredes et al. (2021) | 144 La Liga Footballers (mean age 27) | Prospective cohort | Chronic Match Congestion | Injury | Players with lower match exposure and reduced running distances were more likely to suffer muscle injuries. |
| Waldén, Hägglund et al. (2005) | 266 Footballers playing in UEFA champions league & national team matches (mean age 26) | Prospective cohort | Chronic Match Congestion | Injury | Players who were exposed to national team play had a higher match exposure. However, the higher match exposure did not influence the risk of injury when comparing these players with those who did not have international obligations. |
| Garcia-Romero-Perez, Ordonez et al. (2021) | 29 English Premier League Players (mean age 28) | Prospective cohort over 2 consecutive seasons | Chronic Match Congestion | Health/Wellness | Players who played more than 60 minutes in multi-match weeks had significantly higher pregame creatine kinase levels and increased muscle soreness and fatigue compared to single-match weeks. |
| Saidi, Ben Abderrahman et al. (2020) | 16 Footballers from Tunisian First Division | Prospective cohort over a 12-week competitive period | Chronic Match Congestion | Health/Wellness | Significant hormonal changes (decreased testosterone and increased cortisol) were observed during the congested match period. Psychological parameters like mood state disturbances (e.g., fatigue, tension) were associated with declines in physical fitness. However, hormonal changes did not correlate with fitness performance. |
| Saidi, Zouhal et al. (2022) | 14 Footballers playing in Tunisian First Division (mean age 21) | Repeated measures cohort | Chronic Match Congestion | Health/Wellness | There was a significant increase in inflammation and muscle damage markers (C-reactive protein, creatine kinase) and a decline in physical fitness and wellness status during the congested period. |
| Saidi, Zouhal et al. (2019) | 18 Footballers playing in Tunisian First Division (mean age 20) | Repeated measures cohort over a six-week period of match congestion | Chronic Match Congestion | Health/Wellness | The congested period led to significant declines in physical fitness and changes in haematological parameters, suggesting a need for proper recovery to avoid overtraining. |
| Fuller (2018) | 4 English Premier League teams | Retrospective modelling cohort | Chronic Match Congestion and Break | Injury | Despite playing more matches, successful teams experienced a higher match injury burden but a lower training injury burden because they had fewer training sessions.  The overall injury burden (both match and training injuries) was higher for successful teams compared to unsuccessful teams due to the increased number of competitive matches.  The model indicated that there would be no benefit of adopting a 4-week mid-season break but reducing the number of matches played by reducing the number of clubs competing in the league, or cup fixtures, would help lower the overall injury burden​. |

Table SC2. Rest, recovery and/or fatigue studies

| Study | Sample | Research Design | IMC Indicator | Outcome Measure | Key Findings |
| --- | --- | --- | --- | --- | --- |
| Delaval, Abaidia et al. (2022) | 46 Ligue 1 players (mean age 24) | Retrospective cohort over two seasons (2015-2017) | Acute Fixture Congestion and Rest/Recovery | Injury | During a congested schedule, noncontact injuries were associated with fatigue and muscle soreness, especially 2-3 days after matches. Recovery protocols involving sleep, fatigue, and muscle soreness monitoring, and isometric force tests helped reduce injury risk during congested schedules​. |
| Noor, McCall et al. (2021) | 37 Footballers playing for the Australian National team (mean age 26) | Retrospective cohort | Acute Fixture Congestion and Rest/Recovery | Health/Wellness | During international tournaments, perceived load and recovery profiles were largely influenced by exposure to match play.  Acute match congestion (2 matches within 4 days) impaired players' pre-match perceived status compared to non-congested microcycles but did not exacerbate post-match fatigue and recovery responses |
| Ascensao, Rebelo et al. (2008) | 16 Footballers playing in Portuguese Secondary Division (mean age 21) | Repeated measures cohort | Fatigue | Health/Wellness | Increased oxidative stress and muscle damage markers (creatine kinase, myoglobin, malondialdehyde, etc.) and decreased antioxidant markers (sulfhydryl groups) up to 72 hours post-match. Significant impairments in muscle function were observed during the recovery period. |
| Krustrup, Ørtenblad et al. (2011) | 7 Footballers playing in Danish Second Division | Repeated measures cohort | Fatigue | Health/Wellness | Muscle glycogen levels recovered within 72 hours, but muscle soreness and reduced maximal voluntary contraction force were observed for up to 72 hours post-game​ |
| Rampinini, Bosio et al. (2011) | 20 Serie A Footballers (mean age 19) | Quasi-experimental control period design | Fatigue | Health/Wellness | Fatigue, as measured by maximal voluntary contraction (MVC) and sprint performance, was significantly reduced immediately after a 90-minute match, while short-passing ability remained unaffected.  Central fatigue (reduced voluntary activation and EMG activity) was the primary cause of performance declines, while muscle soreness was linked to peripheral fatigue.  Recovery of all variables to baseline occurred within 48 hours, showing a faster recovery in elite players compared to lower-level athletes |
| Morgans (2014) | 9 players from the English championship (mean age 26) | Repeated measures cohort | Rest/Recovery | Performance and health/wellness | Players did not experience impaired physical match performance in the second match after 75 hours of recovery.  Jump height was reduced post-match 1 for up to 40 hours but returned to near baseline levels by 35 hours post-match 2. |
| Penedo-Jamardo, Rey et al. (2017) | 4496 Bundesliga Players | Prospective cohort | Rest/Recovery | Performance | A reduction in total distance, fast runs, and sprints was observed in recovery cycles of less than four days. |

Table SC3. Midseason break studies

| Study | Sample | Research Design | IMC Indicator | Outcome Measure | Key Findings |
| --- | --- | --- | --- | --- | --- |
| Fuller (2018) | 4 Premier League Teams | Retrospective modelling cohort | Acute and Chronic Match Congestion, and Midseason Break | Injury and Performance | Despite playing more matches, successful teams experienced a higher match injury burden but a lower training injury burden because they had fewer training sessions.  The overall injury burden (both match and training injuries) was higher for successful teams compared to unsuccessful teams due to the increased number of competitive matches.  The model indicated that there would be no benefit of adopting a 4-week mid-season break, but reducing the number of matches played by reducing the number of clubs competing in the league, or cup fixtures, would help lower the overall injury burden​. |
| aus der Funten, Faude et al. (2014) | 372 1^st^ and 2^nd^ Bundesliga Footballers | Prospective cohort | Midseason Break | Injury | Shortening the winter break from 6.5 to 3.5 weeks did not affect overall injury incidence but was associated with an increased number of training and knee injuries, and a trend towards more severe injuries (time loss > 7 days)​ |
| Ekstrand, Spreco et al. (2019) | 56 Teams Playing in UEFA Competitions and top tier domestic leagues | Prospective cohort over seven seasons | Midseason Break | Injury | Teams without a winter break (e.g., English teams) lost 303 more player-days due to injuries than teams with a winter break.  The absence of a winter break was associated with a higher incidence of severe injuries and greater injury burden.  Having a winter break had no significant effect on team training attendance or match availability. |
| Jamil, McErlain-Naylor et al. (2020) | 3494 match observations from Bundesliga, Ligue 1, La Liga and Premier League | Retrospective case control | Midseason Break | Performance | A winter break longer than 13 days negatively impacted technical performance in the German Bundesliga and French Ligue 1, while shorter breaks (less than 13 days) or no break had no significant effect on performance. |
| Rodriguez-Fernandez, Sanchez-Sanchez et al. (2018) | 17 La Liga Footballers (average age: 24) | Repeated measures cohort | Midseason Break | Performance | The 2-week in-season break led to a decrease in repeated-sprint ability (RSA) with worsened best time (RSAbest), mean time (RSAmean), and total time (RSAtotal). The detrimental effects were more pronounced in faster players. However, the break did not affect intermittent endurance performance​ |

Table SC4. Travel studies

| Study | Sample | Research Design | IMC Indicator | Outcome Measure | Key Findings |
| --- | --- | --- | --- | --- | --- |
| Gilbert, Dixon et al. (2020) | 978 MLS Games | Retrospective cohort | Travel | Performance | A negative impact was found on visiting teams' performance when they incurred a connection during air travel and when traveling eastward.  No significant effects were observed from other travel variables such as the number of hours travelled, or time zones crossed. |
| Zacharko, Konefal et al. (2022) | 340 Players at World Cup (average age: 27) | Retrospective cohort | Travel | Performance | Players traveling in the same zone, or from East to West performed better in terms of total distance covered and number of passes compared to those traveling West to East. The direction of time-zone travel significantly affected physical and technical performance, influencing tournament rankings. |
| Fowler, McCall et al. (2017) | 22 Australian National Team Players competing at World Cup | Retrospective cohort | Travel | Health/Wellness and Performance | The study observed significant effects of long-haul travel on sleep, jetlag, and wellness. Players experienced reduced sleep duration during travel and heightened jet-lag post-arrival, with significant effects persisting for four days. Training loads decreased, and wellness scores also dropped following travel​ |
| Fowler, Duffield et al. (2015) | 16 Footballers competing at Australian A-League and Asian Champions League (average age: 27) | Retrospective cohort | Travel | Performance | Sleep was reduced the night before travel and after competition. Subjective jet lag was higher after travel, but this may have been interpreted as fatigue from competition rather than long-haul travel itself, across 1 time zone. No major negative impact of the travel was found on player preparedness​ |
| Clements, Ehrmann et al. (2023) | 68 Australian National Team Players | Retrospective cohort | Travel | Health/Wellness | Jet lag ratings were highest for travel from Europe to Australia and Asia to Europe.  Daytime arrivals resulted in better perceptual responses compared to morning or late-night arrivals.  Players reported worse perceptual outcomes (e.g., fatigue, sleep, soreness) when traveling back to their club compared to traveling to the national team.  Perceived jet lag was more responsive to travel demands than other wellness measures​ |
| Fullagar, Duffield et al. (2016) | 15 International Footballers (average age: 26) | Retrospective cohort | Travel | Performance | Players experienced poor sleep during long-haul travel, and around match nights. Sleep increased significantly on the night following the arrival. Therefore, rest and recovery is recommended for at least 24 hours after long-haul international travel, and matches. |
| den Hollander, Kerkhoffs et al. (2024) | 860 Footballers playing in top 5 European Leagues (average age: 26) | Retrospective case-control over two competitive seasons (2021-2023). | Acute and Chronic Match Congestion, and Travel | Injury | Injured players had higher match workloads and travelled further across more time zones compared to non-injured players. |
| Clements, Ehrmann et al. (2023) | 50 International Footballers ((average age: 26) | Retrospective cohort over 3 years (2018–2021) | Travel | Health/wellness | Perceptual ratings of fatigue, sleep, and soreness worsened as time zone changes increased, especially for eastward travel. Traveling across 9+ time zones resulted in significantly worse perceived fatigue, sleep, and wellness scores on days 1 and 2 post-arrival compared to shorter trips. Eastward travel also led to poorer sleep ratings compared to westward travel​. |
| Lastella, Roach et al. (2019) | 25 Footballers playing in the AFC Champions League | Retrospective cohort | Rest/Recovery and Travel | Health/wellness | Players' sleep/wake behaviours were disrupted during flights, with players getting 3.6 hours less sleep during flights compared to at home or away.  Travel and competition schedules significantly affected sleep, resulting in poorer recovery for the athletes. |

Table SC5. Survey studies

| Study | Sample | Research Design | IMC Indicator | Outcome Measure | Key Findings |
| --- | --- | --- | --- | --- | --- |
| Liporaci, Yoshimura et al. (2021) | 100 Footballers playing in the 1^st^ to 4^th^ Brazilian leagues (mean age 25) | Cross-sectional survey | Acute and Chronic Fixture Congestion | Injury | Top five risk factors for injuries perceived by players included short intervals between matches and a high number of matches in a season. |
| Pillay, Burgess et al. (2022) | 1055 Players across multiple countries globally (mean age 27) | Cross-sectional survey | Acute and Chronic Fixture Congestion, Rest/Recovery, and Travel | Injury and Performance | 42% of players indicated that back-to-back matches should be limited to three. 69% of respondents felt that their off-season and in-season breaks were infringed upon by club or national team obligations. 83% of the players believed there should be regulations to allow sufficient rest periods to avoid excessive workload. 52% of the players indicated that the congested match schedule negatively affected their mental health, largely due to insufficient recovery time. 55% of respondents believed they had suffered an injury due to match congestion, and 20% believed they had sustained multiple injuries due to the packed schedule. Players reported that long-distance travel exacerbated the issues of recovery and rest, especially when traveling between matches in a congested schedule. The demands of international and domestic competitions required frequent long-distance travel, further reducing recovery time. |
| Field, Harper et al. (2021) | 80 Practitioners from multiple elite clubs and national teams globally | Cross-sectional survey | Acute Fixture Congestion and Rest/Recovery | Performance | Extra rest days were used by 94% of teams, mainly implemented on match day +1 (MD+1)​.  Fixture congestion was recognized as a factor affecting recovery, with players sometimes competing in two or more matches per week. In such cases, rest and recovery were prioritized to mitigate the impact of fixture congestion​. |
| Gouttebarge, Brink et al. (2019) | 543 Players across multiple countries globally (mean age 26) | Cross-sectional survey | Chronic Match Congestion, Break, Rest/Recovery, and Travel | Injury | 35-40% of players reported playing too many matches with insufficient recovery.  60% stated that long-distance travel negatively impacted their performance and health.  Players favoured a 14-day in-season break and a 5-week off-season break. |
| McCall, Carling et al. (2014) | 44 Teams from top division Leagues | Cross-sectional survey | Fatigue | Injury | Second highest risk factor for non-contact injuries was fatigue. |
| McCall, Dupont et al. (2016) | 33 Teams participating in UEFA competitions and domestic leagues | Cross-sectional survey | Fatigue | Injury | Accumulated fatigue was as one of the most important risk factors for injury |
| Di Salvo, Bonanno et al. (2023) | 300 Footballers playing in top division UEFA domestic leagues | Cross-sectional survey | Fatigue and Travel | Performance | Players reported significant post-match fatigue, which was exacerbated by away match travel. Fatigue was influenced by travel distances and match intensity, highlighting the importance of recovery interventions after away games. |
