# Supplementary material for: The International Match Calendar in Men's Professional Football: An Expert Position Statement
Source: Scand J Med Sci Sports. 2025 Nov 6;35(11):e70163. doi: 10.1111/sms.70163 (PMC12591028; doi:10.1111/sms.70163)
Supplement: Supplementary file 4 — Data S4: sms70163‐sup‐0004‐Supinfo4.pdf. [file SMS-35-e70163-s004.pdf]

## Supplemental file D

### Statements

1. **There should be a maximum limit of 45 matches played (club and national team) per player per season.**

| % Agree | % Disagree | % Not enough Research | % Disagree Completely | % Different Number | Min Number | Max Number | Median Number |
|---|---|---|---|---|---|---|---|
| **25%** | **75%** | 25% | 31% | 18% | 3 | 65 | 55 |

*Agree there should be a limit – 25%+25%+18% = 68%*

***Feedback***

*Too many unknowns*

there are too many variable to make sweeping statements like the above.

Many other factors such as player position, playing style, age, injury history etc come into this decision

Research is key and integral to decision making on the subject.

*Focus on Minutes Played*

It is difficult to answer those questions as I suppose the number of minutes should be consdered instead.

The absolute thresholds of involvement in the number of events/matchdays doesnt consider the distribution of playing time

The load needs to be specific to minutes played. No issue with players being included in games frequently where minutes are lower or players don't play

Many of the statements do not make sense to apply to appearances only - total exposure in minutes needs to be considered.

*Should be Managed on an individual/club basis*

this is always going to be experiential and anecdotal as some players will be able to cope depending upon multiple factors. however this guidance will help to manage the load

Rules shouldn't be put in place for this. Internally staff need to be better at managing player load

Always depends on the individual constitution

I think it is the responsibility of the club to know what a player is capable of performing.

*Other*

Lets not ignore the number of players who avoid injury still outweighs the number who get injured.

I believe, generally, that restrictions should be placed on matches played, rather than squad inclusions or appearances.

analyse cumulative load between international and club games time to recover between international break and games with club

***Rephrase***

Keep positive (>66% agree there should be a limit) without specifying number.

***New Statement***

There should be a maximum limit of matches played (club and national team) per player per season.

**2. There should be a distinction between the maximum number of matches played, appearances, and squad inclusions a player can make for their club and for their national team.**

| % Agree | % Disagree | % Not enough Research | % Disagree Completely |
|---|---|---|---|
| **67%** | **33%** | 7% | 25% |

***Rephrase***

Rephrase to focus on minutes played.

***New Statement***

There should be a distinction between the maximum number of matches and/or minutes a player can make for their club and for their national team per season.

**3. There should not have more than 6 consecutive back-to-back squad inclusions.**

| % Agree | % Disagree | % Not enough Research | % Disagree Completely | % Different Number | Min Number | Max Number | Median Number |
|---|---|---|---|---|---|---|---|
| **31%** | **69%** | 11% | 55% | 4% | 9 | 10 | 9.5 |

*Agree there should be a limit – 31%+11%+4% = 45%*

***Feedback***

Difficult to put a limit on games due to games where a player is included. You would be better at looking at making sure turnover between games is sufficient. You may also be better to increase the number of subs used - going to 5 subs from 3 has significantly reduced players load

Minutes played in each match is important ie 6 consecutive 90 minutes is very different from a mixture of being substituted on or off.

In my opinion 8 back-to-back appearances of more than 60 minutes during one month is a maximum. But more importantly, I strongly believe that the duration of the off-season and mid-season break to recover completely is more important than the number of games. But indeed a maximum of back-to-back appearances (with midweek and weekend matches) would be preferable as well, certainly for players who play in September, October and November with their national teams.

i think we (medical/sports science, even fans & media) understand that being exposed to multiple game weeks & playing high amounts of minutes in those games, will increase the risk of injury. However, I dont think we know or can give a specific number which is the maximum that should be allowed.

***Rephrase***

Rephrase into negative (<67% agree there should be a limit) without specifying number.

***New Statement***

There should not be a limit on the number of consecutive back-to-back squad inclusions.

**4. Players should be monitored by the club during the off-season break**

| % Agree | % Disagree | % Not enough Research | % Disagree Completely |
|---|---|---|---|
| **67%** | **33%** | 4% | 29% |

*% Agree there should be monitoring – 71%*

***Feedback***

*Should be monitored*

Including HRV and perhaps psychological stages (mental health related)

For player benefit

Difficulty in practically applying this monitoring process with the majority of current technologies available. This can help ensure a more gradual and individualized management to preseason

Surely, this is already being done.

Helps bridge into pre-season

Should be monitored but have sufficient time away from the club - this is to ensure players health is looked after

*Should be optional*

technical and medical management is the responsibility of the club and must be offered to the player. However, the player must have the right to refuse during the off season break

A carefull bamlance need to be considered by club to respect privacy outside training ground. Therefore mutual agreement should be consdiered between players and club to allow such practices. While following training program is important (if long time break),monitoring might lead to some ethical considerations.

i think teams should help offer support to players (by means of a programme to follow) if the players request that & i think staff are obliged/under pressure to do that as well. but in terms of tracking (wellness, GPS) my preference has always been to give them space and track them when they return.

Players should be expected to have a certain level of health & fitness following off season break however club interaction should be at the players discretion

That's doesn't need to be monitoring a training programme but feel there should be some guidance on recovery. Some player may need some down time but feel pressure to keep training guidance to help find balance when recovery is needed maybe important for some but don't think they need daily contact they need time away.

Because we perform a whole year, i think you should let the players free. Of course is the possiblity there if the player would like it.

*No monitoring*

The pleasure should not "monitored" or should be given programs and guidance and then left to their own devices. If pies are not independent thinking enough to do this during the off-season it is the education of the players, which is the problem, not the monitoring.

I think that during this break the club must leave the player completely free to rest. On the other hand, they can set up a program to submit to the players so that they respect it but without monitoring itmonitored yes. however rest is the most important part of the off season

Players will benefit from not being monitored during their holidays

Some players prefer sensible non-intrusive advice/programming during the off season.

I think the players need a complete break without interference of the club - they should completely disconnect (from their club, but ideally from football in general) (to improve mental health)

I think players need time away. However, they should still have access to programs.

Competent adult players should be allowed and given the responsibility to follow the prescribed individualised  off season plan without monitoring

I think respecting the players "off time" is important. they are professional athletes and need some privacy

Players should be able to shut off from all commitments in order to facilitate mental and cognitive recovery

*Combination of Monitoring & Black-out period*

but also having a period off completely with no or very little comms

The club should oversee the graded return back with a compulsory period of rest. E.g. 2 weeks rest then 2 week period which involves them doing no more than 7-8 sessions.

*Other*

High level multi center research is integral to on this subject.

***Rephrase***

Rephrase that it should be optional, with the focus on the club not the player.

***New Statement***

Clubs should provide players with optional monitoring (e.g. following a prescribed training plan or wellness monitoring) during the off-season break.

5. **There should be a minimum of two days between a long-haul flight and a subsequent squad inclusion to adequately recover from either travel fatigue or jet lag**

| % Agree | % Disagree | % Not enough Research | % Disagree Completely | % Different Number | Min Number | Max Number | Median Number |
|---|---|---|---|---|---|---|---|
| **69%** | **31%** | 7% | 18% | 5% | 1 | 7 | 1 |

*% Agree there should be a rest period – 69%+7%+5%= 81%*

***Feedback***

*Agree*

*common sense and evidence*

*Especially when travelling eastwards in long-haul flights.*

*Player recovery is vital to performance*

*Disagree*

*Every players involvement should be taken in context.*

*Research shows the differences in those who travel more become more adaptable to it*

*No prescription should be given. Decisions on this should be made on an individual basis by highly skilled and experienced practitioners.*

*This woudl mean to play for some national teams players would have to miss an extra club game.*

*This cannot be a rule as there will always be exceptions.  I agree with the principle and clubs should not be force to play a fixture within two days of a long-haul flight.*

*Jet lag and travel fatigue may not necessarily incur negative outcomes for all players on each occasion. More appropriate and sensitive monitoring techniques are alternatively recommended*

***Rephrase***

Keep positive (>66% agree there should be a rest period) without specifying number.

***New Statement***

There should be a rest period between a long-haul flight and a subsequent squad inclusion to adequately recover from either travel fatigue or jet lag.

**6. There should be specific workload safeguards for young players (under 21 years old).**

| % Agree | % Disagree | % Not enough Research | % Disagree Completely |
|---|---|---|---|
| **73%** | **27%** | 11% | 16% |

*% Agree there should be specific safeguards – 73%+11%= 84%*

***Feedback***

*Should only be at U18/U19*

The club that I currently work at we have an average age of 22.3 years with most of our players below the age of 21 as we have a 30 year old player swaying the upper age. The vast majority of them have been involved in the majority of our fixtures this season either as a substitute or in the starting 11. If they have their load managed correctly then I disagree with the statement. Under 19s players I do believe should have specific workload safeguards due to maturation.

too much variation to be definitive. would suggest U19

I think it should be more specific to under 18 and then adapted to players on their maturation status.

...to an extent. Maybe U18, but not U21. And again, I think it should be in teh hands of experienced and qualified practitioners - not put in rule.

*Too many unknowns*

too difficult to benchmark workload levels beyond number of matches and match exposure, similar to their elder peers

But we need to back this statement with science in the near future

i don't think we have good research to tell us what the age cut-off should be.  for men ? 18 or 19 or 20? for women maybe it's a different age?

We do not have sufficient evidence or knowledge to know what this may look like

*Agree*

With ensuring an efficient and secure reporting system.

It me be suprising but I suppose, those workload  safeguards should be apply in both way:  - Over exposure to match etc leading to potentially too much fatigue and increased injury risk - Sub exposure where a plauer does not have the oppportunity to train adequatly which might affect is long term development which is equally bad...

This may help manage the top young players who are regular first team starters at that age

*Other*

What are you using to quality work load? Training time/distance covered number of training sessions. Manipulating any of these has very different outcomes for an individual.

***Rephrase***

Rephrase that specific safeguards are necessary but more research is needed.

***New Statement***

More research is needed to decide whether there should be specific workload safeguards for young players (under 21 years old).
